# Supplementary material for: High-throughput protein characterization by complementation using DNA barcoded fragment libraries
Source: Mol Syst Biol. 2024 Oct 7;20(11):5. doi: 10.1038/s44320-024-00068-z (PMC11535334; doi:10.1038/s44320-024-00068-z)
Supplement: Supplementary file 1 — Appendix [file 44320_2024_68_MOESM1_ESM.pdf]

Appendix for: High-throughput protein characterization by complementation using DNA barcoded fragment libraries

Bradley W. Biggs[1], Morgan N. Price[1], Dexter Lai[2], Jasmine Escobedo[2], Yuridia Fortanel[2], Yolanda Y. Huang[1], Kyoungmin Kim[2], Valentine V. Trotter[1], Jennifer V. Kuehl[1], Lauren M. Lui[1], Romy Chakraborty[1], Adam M. Deutschbauer[1,3], Adam P. Arkin[1,2]*

[1]Environmental Genomics and Systems Biology Division, Lawrence Berkeley National Laboratory, Berkeley, CA 94720, USA
[2]Department of Bioengineering, University of California-Berkeley, Berkeley, CA 94720, USA
[3]Department of Plant and Microbial Biology, University of California-Berkeley, Berkeley, CA 94720, USA

*Author to whom correspondence should be addressed; aparkin@lbl.gov

Table of Contents

Appendix Note……………………………………………………………………………………………..pg. 3

Appendix Figures………………………………………………………………………………………pg. 4-26

Appendix Tables…………………………………………………………………………………….......pg. 27-36

Appendix References………………………………………………………………………………pg. 37

Appendix Note

As a potentially helpful summary, we provide this note. Across the different categories of the manuscript, we provided first experimental evidence for function of 53 proteins, of which 42 would have been reasonably expected to perform the identified function owing to their at least 40% amino acid homology to a protein with experimentally verified function. To assign this classification, we assumed that all *Escherichia coli* and *Bacillus subtilis* proteins are already characterized, although some do not exist in curated databases (e.g. ThrB from *B. subtilis*). Similarly, we excluded proteins that we previously identified by way of mutant phenotypes(Price *et al*, 2018), of which there were six. Lastly, we compared to PaperBLAST's database of characterized proteins to ensure that there were no other characterized proteins(Price & Arkin, 2017). This includes two genes that were very close to a previously characterized protein (AAFF19_12795 is 92% identical to Ac3H11_2452, which is the MetZ discussed in the text, and TK06_RS20265 is 92% identical to Q3KK58 of *Pseudomonas aeruginosa*, which is characterized as TrpA).

Together, the complementation data contributed to five diverged enzymes being added to GapMind(Price *et al*, 2020) (BT2186/BT_RS11065 of *Bacteroides thetaiotaomicron* VPI-5482 as AroA, AAFF19_05770 of *Acidovorax sp*. FHTAMBA as HisC, AAFF35_11135 of *Pedobacter sp*. FW305-3-2-15-E-R2A2 as ProB, LRK54_RS01680 of *Rhodanobacter denitrificans* FW104-10B01 as TrpA, and BSU_32240 of *B. subtilis* 168 as ThrB). Finally, broadly speaking our assay identifies proteins capable of completing the missing function, which give significant insight into their own function. However, because of the uniqueness of overexpression in a heterologous host, compared to a potentially lower or context dependent expression in a native host, it is possible that this assay uncovers non-native function, and the putative cystathionine gamma-lyase from *R. denitrificans* FW104-10B01 found in the context of Δ*metB* may be an example.

Appendix Figures

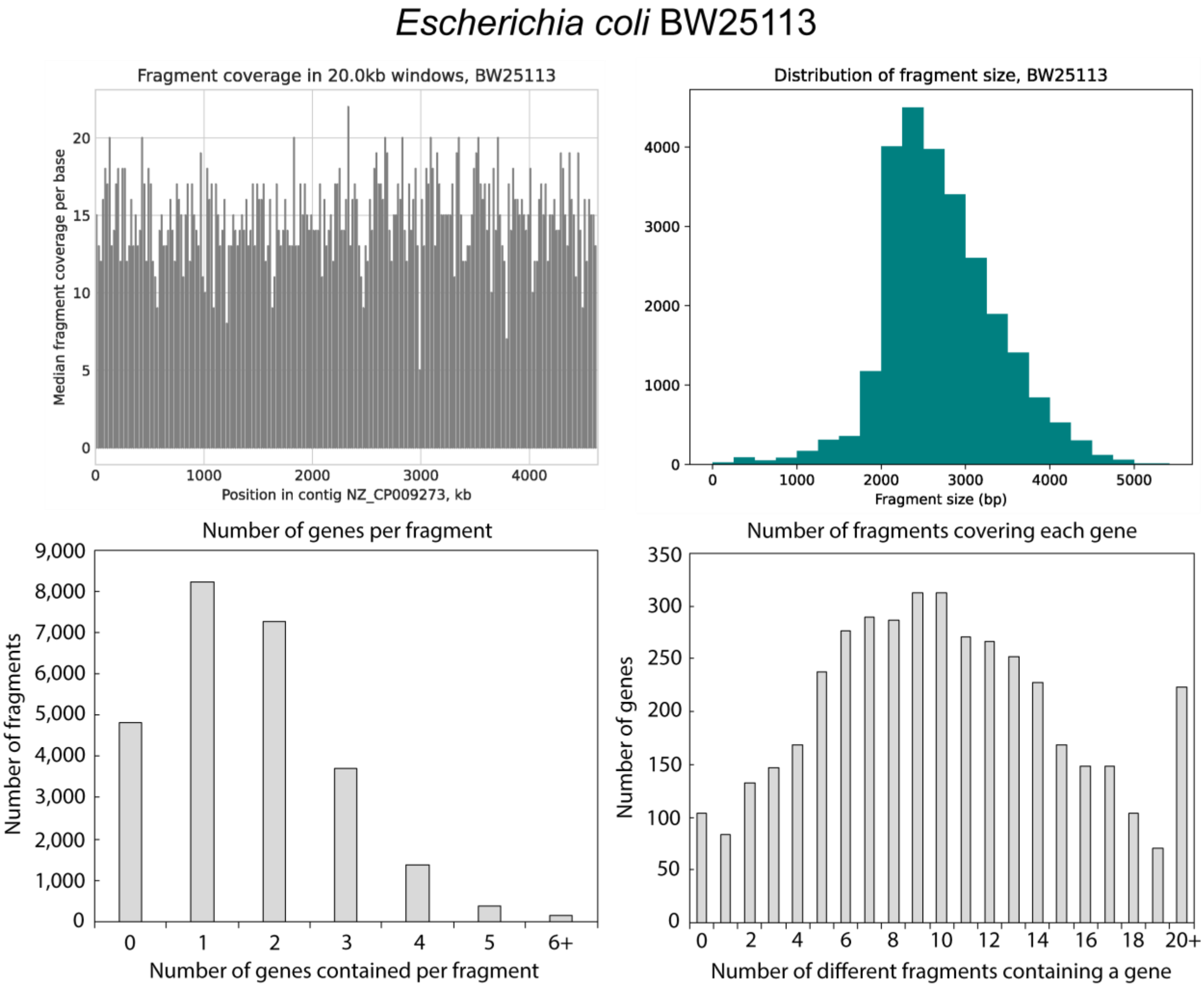

Appendix Figure S1. *Escherichia coli BW25113 fragment library quality figures. For Figure S1 through Figure S11, the top left panel shows a plot of the fragment coverage across the genome. Specifically, the plot shows counts for the number of fragments that give coverage of a region of the genome, with a 20 kb window. The greater the height of the bar, the greater the number of fragments that cover that region of the genome. If there is no bar for a region, no fragment covered it. The top right panel shows the distribution of fragment length. An average of 3 kb sheared genome fragments was sought, but all the distributions show an average below this, with fragments both above and below the target size. The mean is typically closer to 2 kb than 3 kb. The bottom left panel shows the number of genes contained per fragment. The bottom right panel shows the number of independent fragments covering each gene in the genome.*

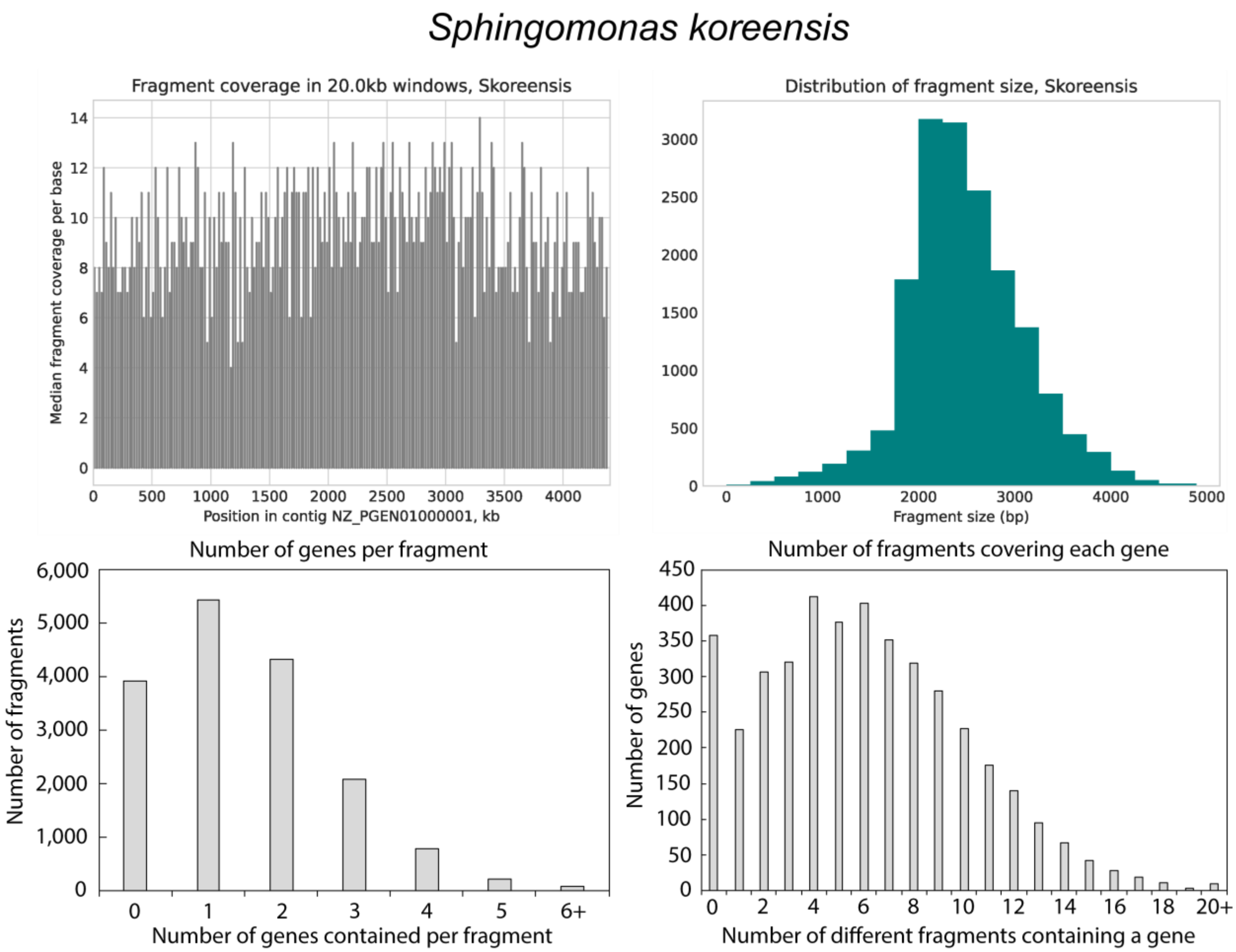

Appendix Figure S2. *Sphingomonas koreensis (JSS26, DSMZ 15582) fragment library quality figures.*

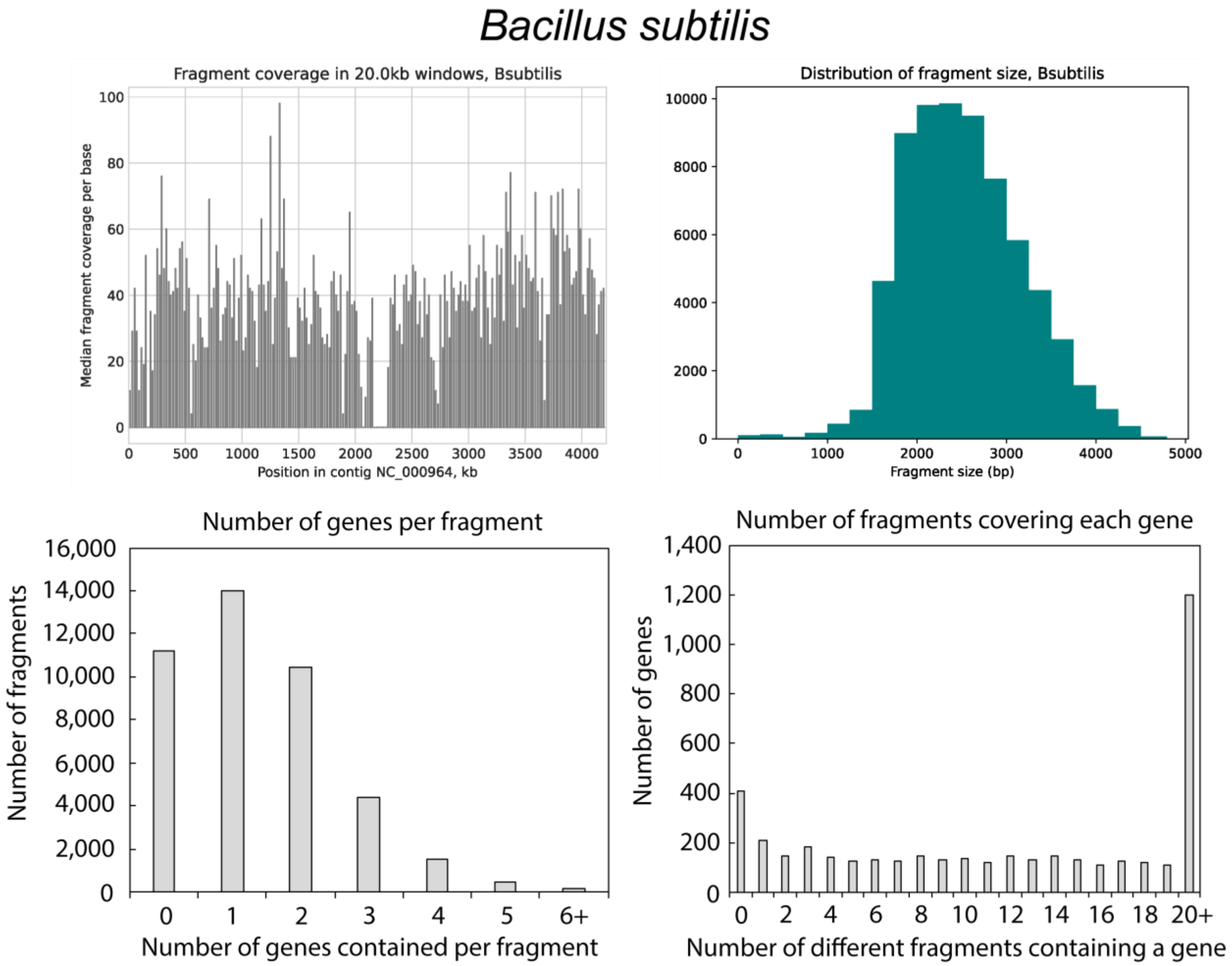

Appendix Figure S3. *Bacillus subtilis fragment library quality figures.*

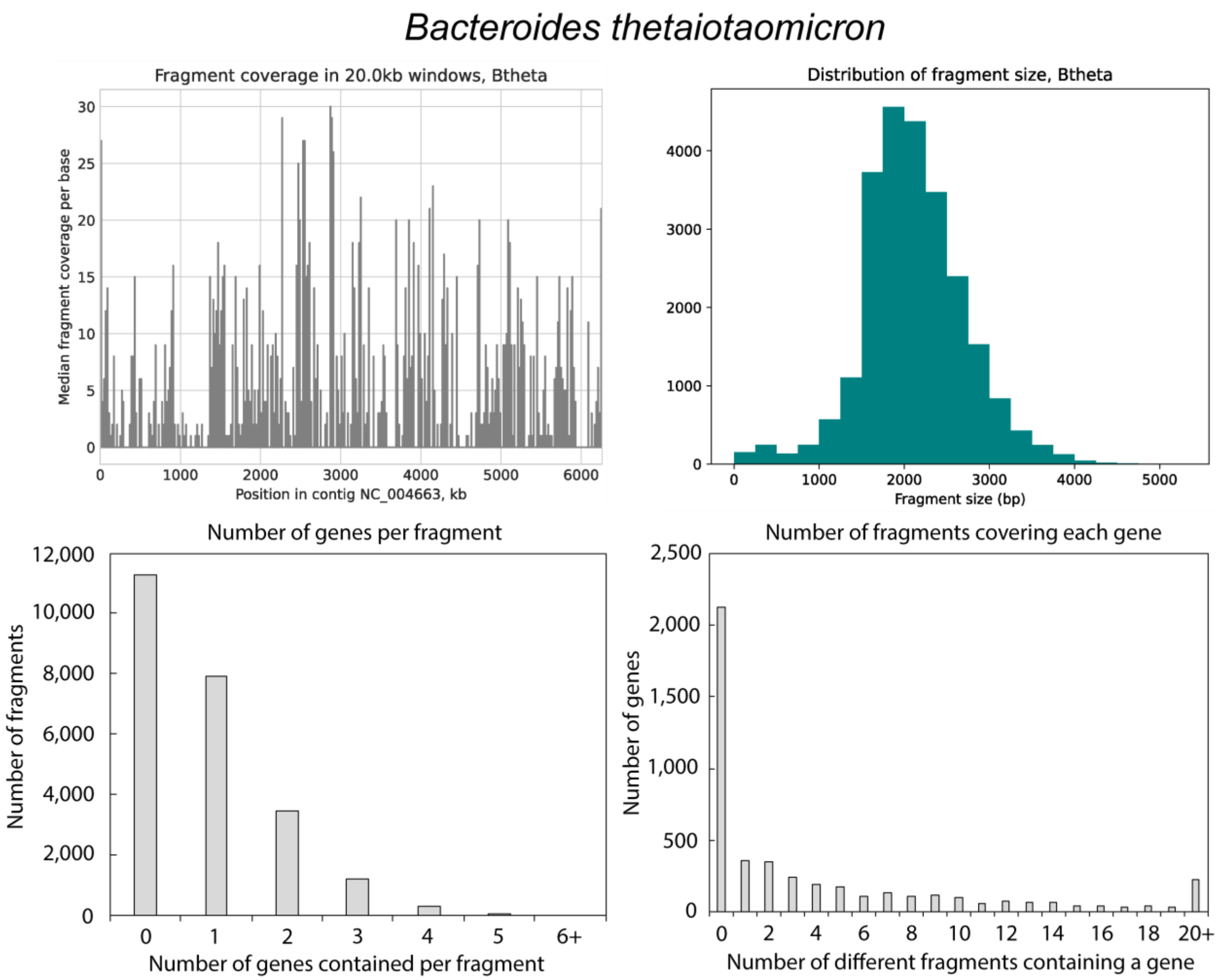

Appendix Figure S4. *Bacteroides thetaiotaomicron fragment library quality figures.*

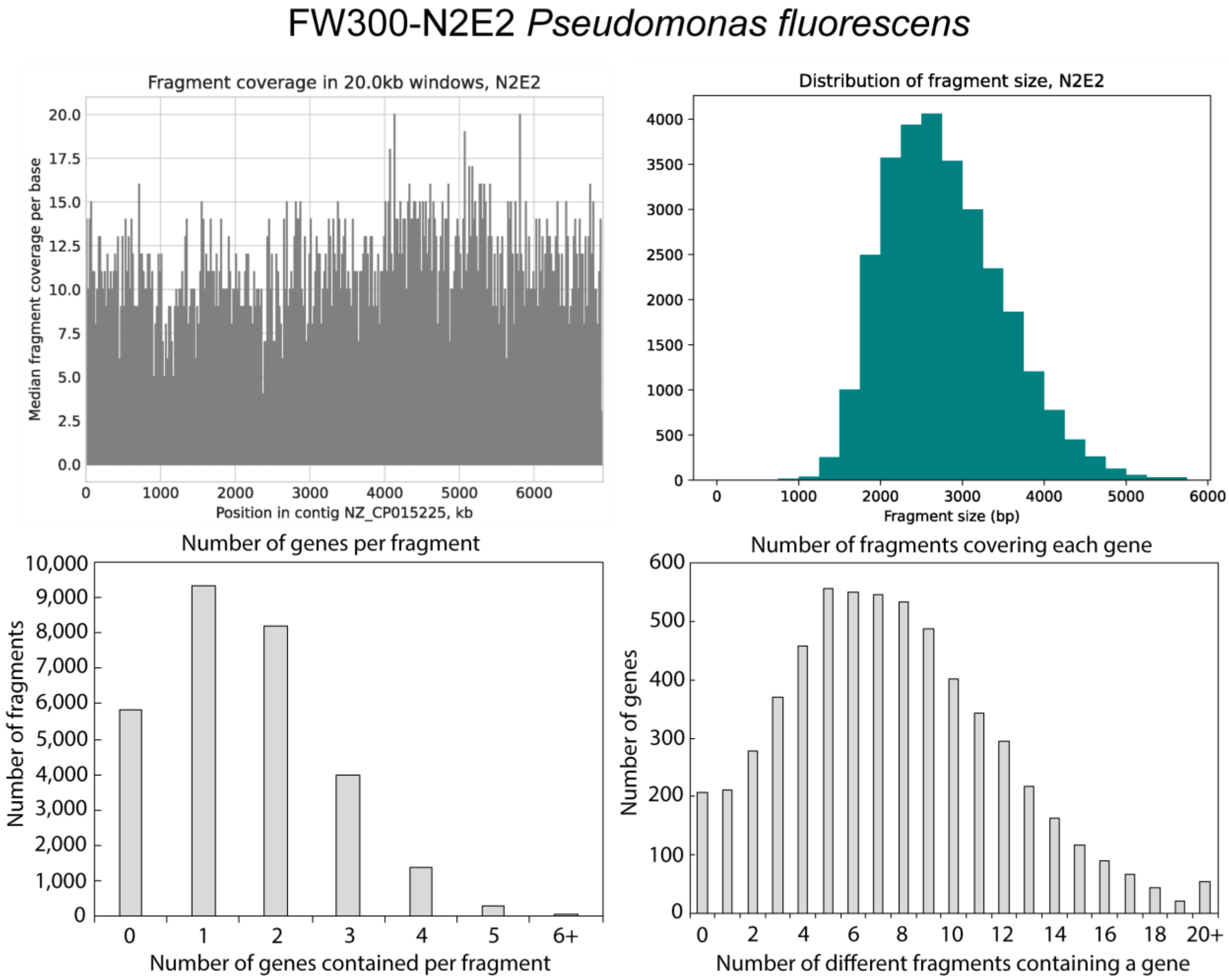

Appendix Figure S5. *FW300-N2E2 Pseudomonas fluorescens fragment library quality figures.*

FW306-1B-D06B *Lysobacter sp.*

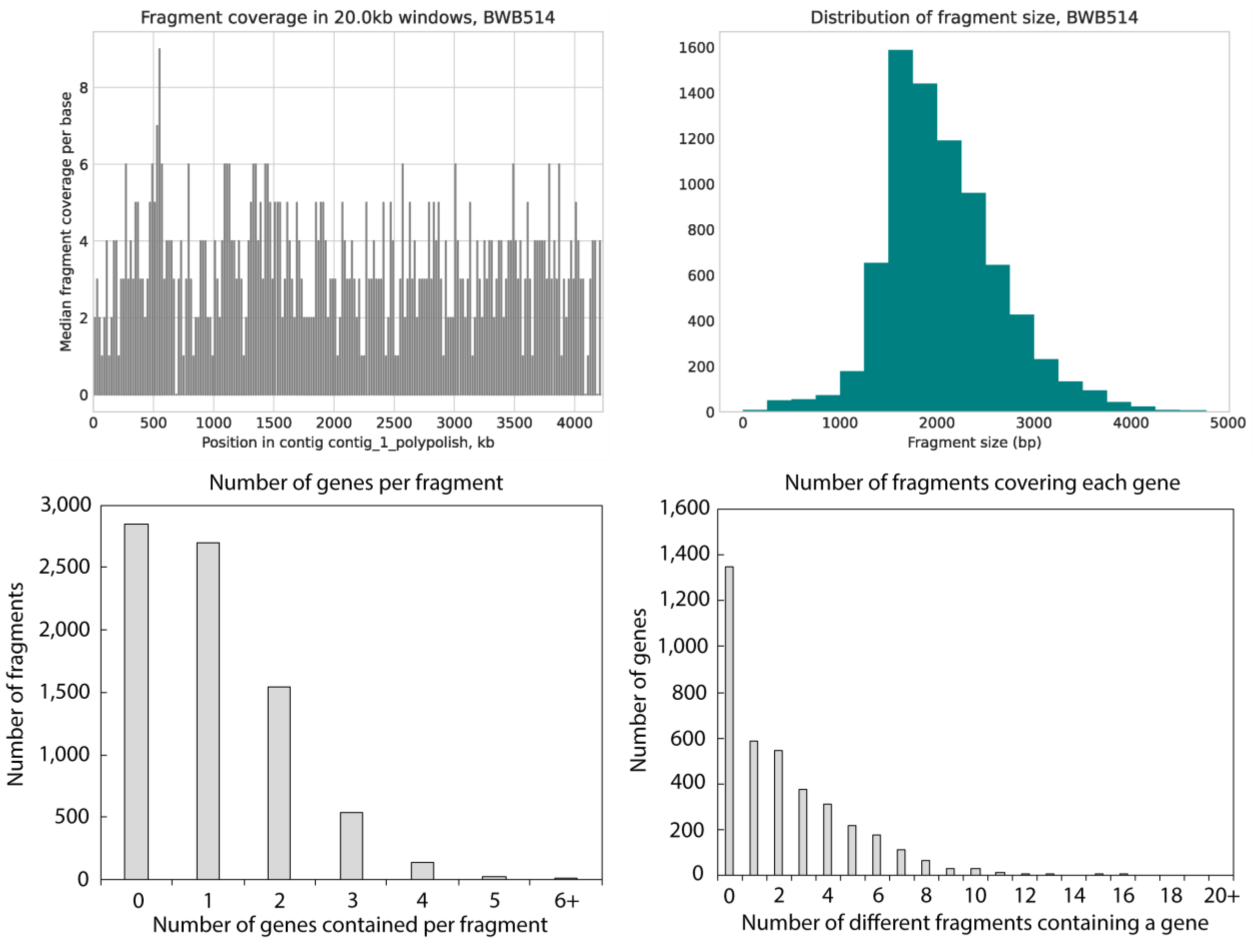

Appendix Figure S6. *FW306-1B-D06B Lysobacter sp. fragment library quality figures.*

GW821-FHT01B05 *Xylophilus sp.*

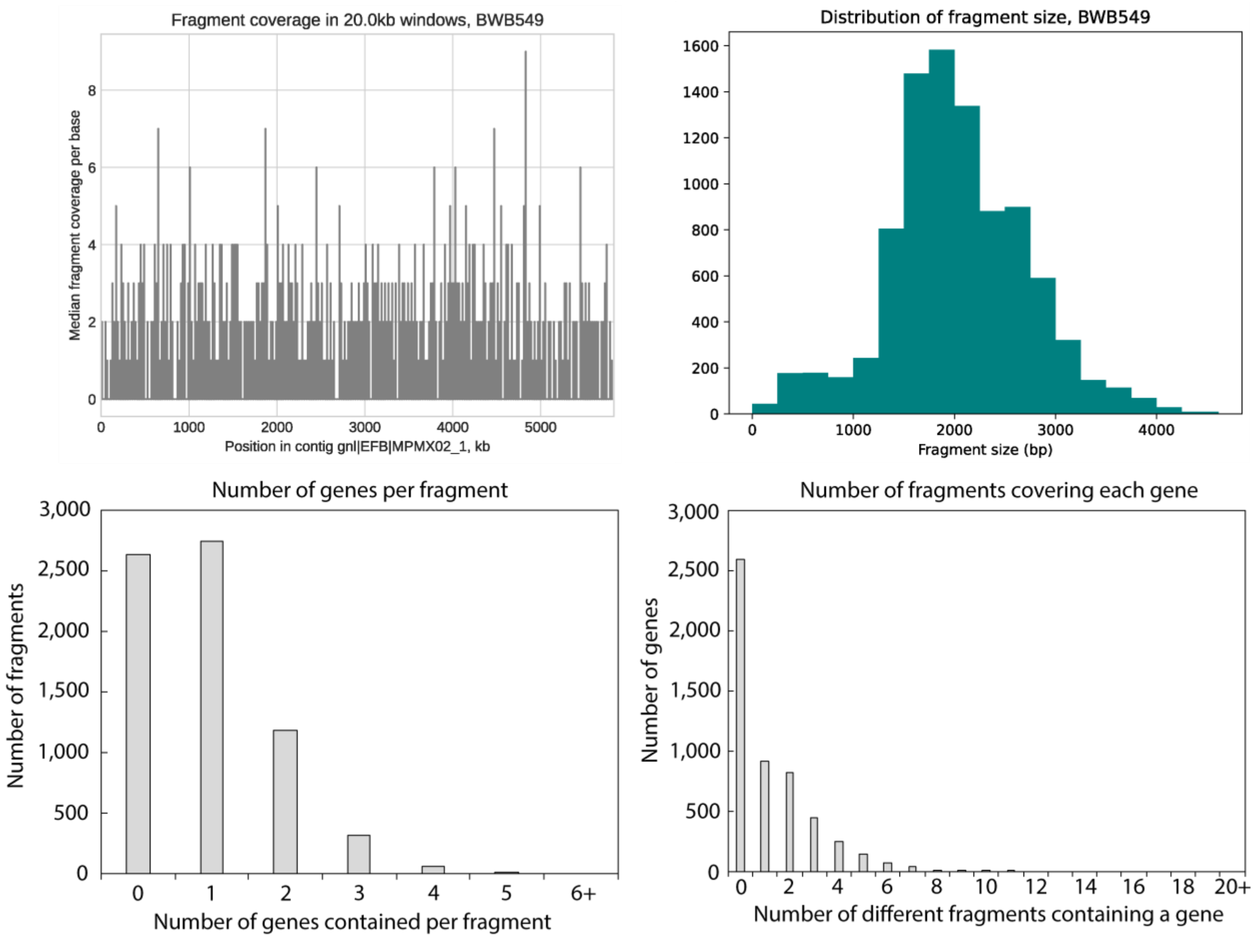

Appendix Figure S7. *GW821-FHT01B05 Xylophilus sp. fragment library quality figures.*

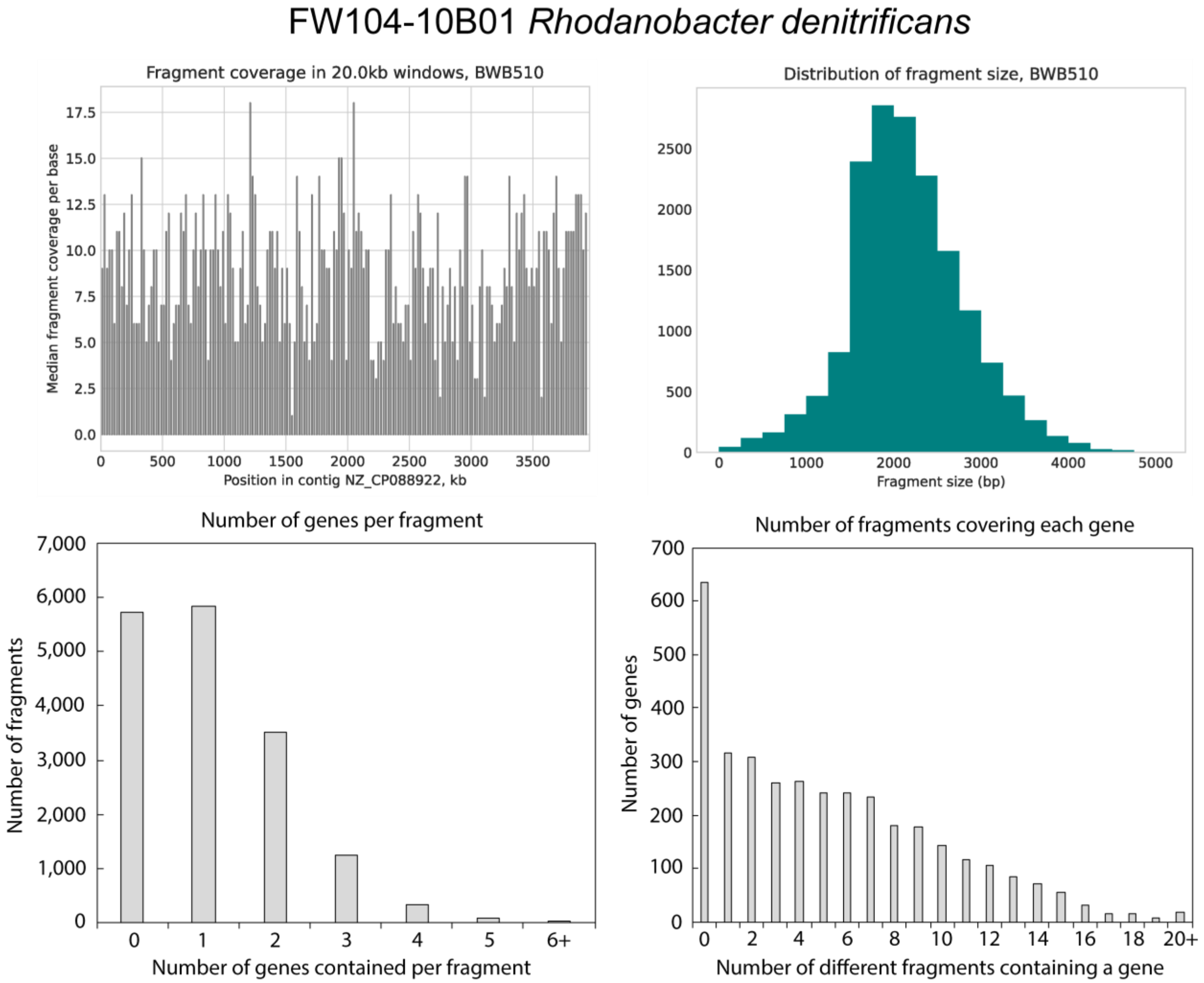

Appendix Figure S8. *FW104-10B01 Rhodanobacter denitrificans Fragment library quality figures.*

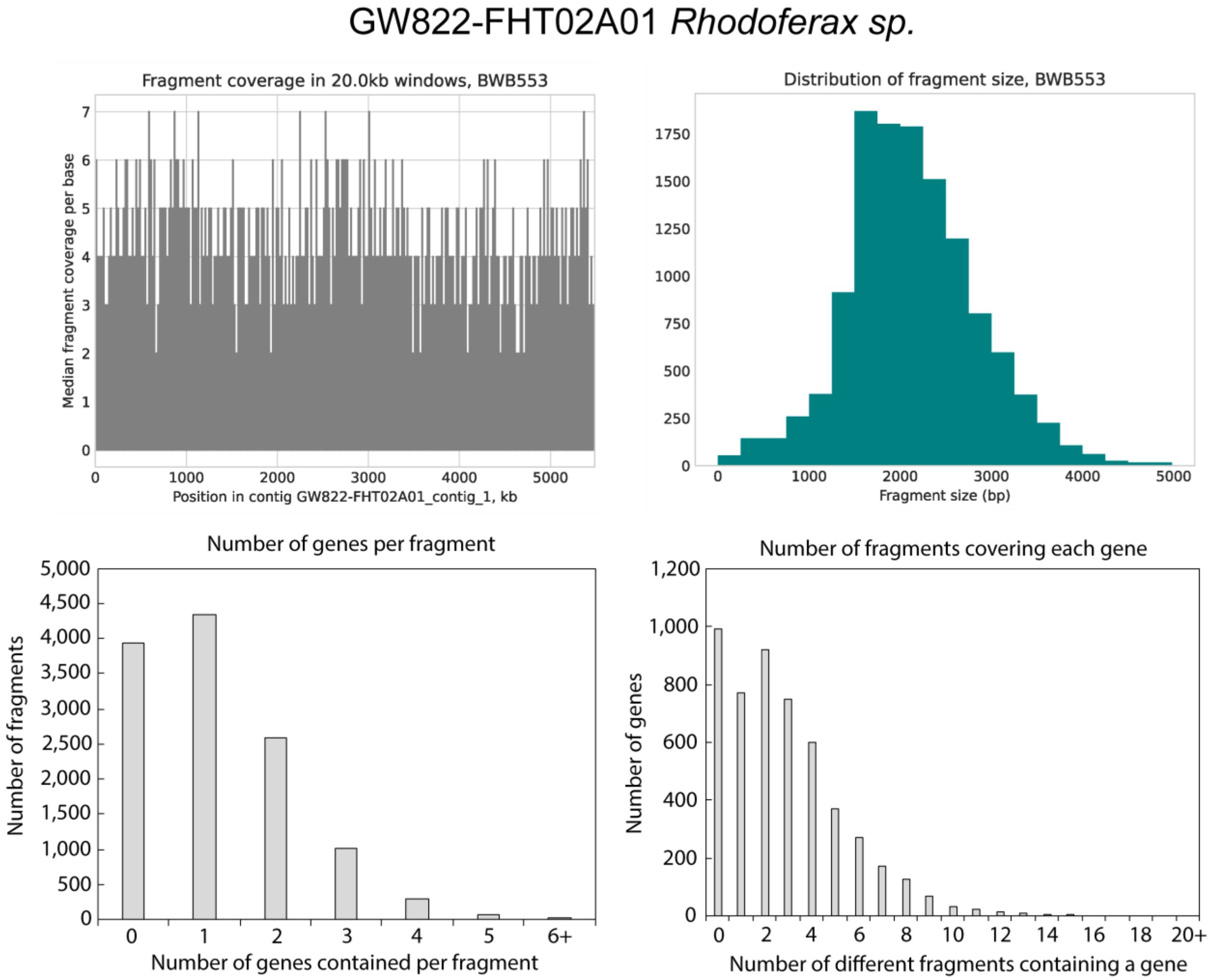

Appendix Figure S9. *GW822-FHT02A01 Rhodoferax sp. fragment library quality figures.*

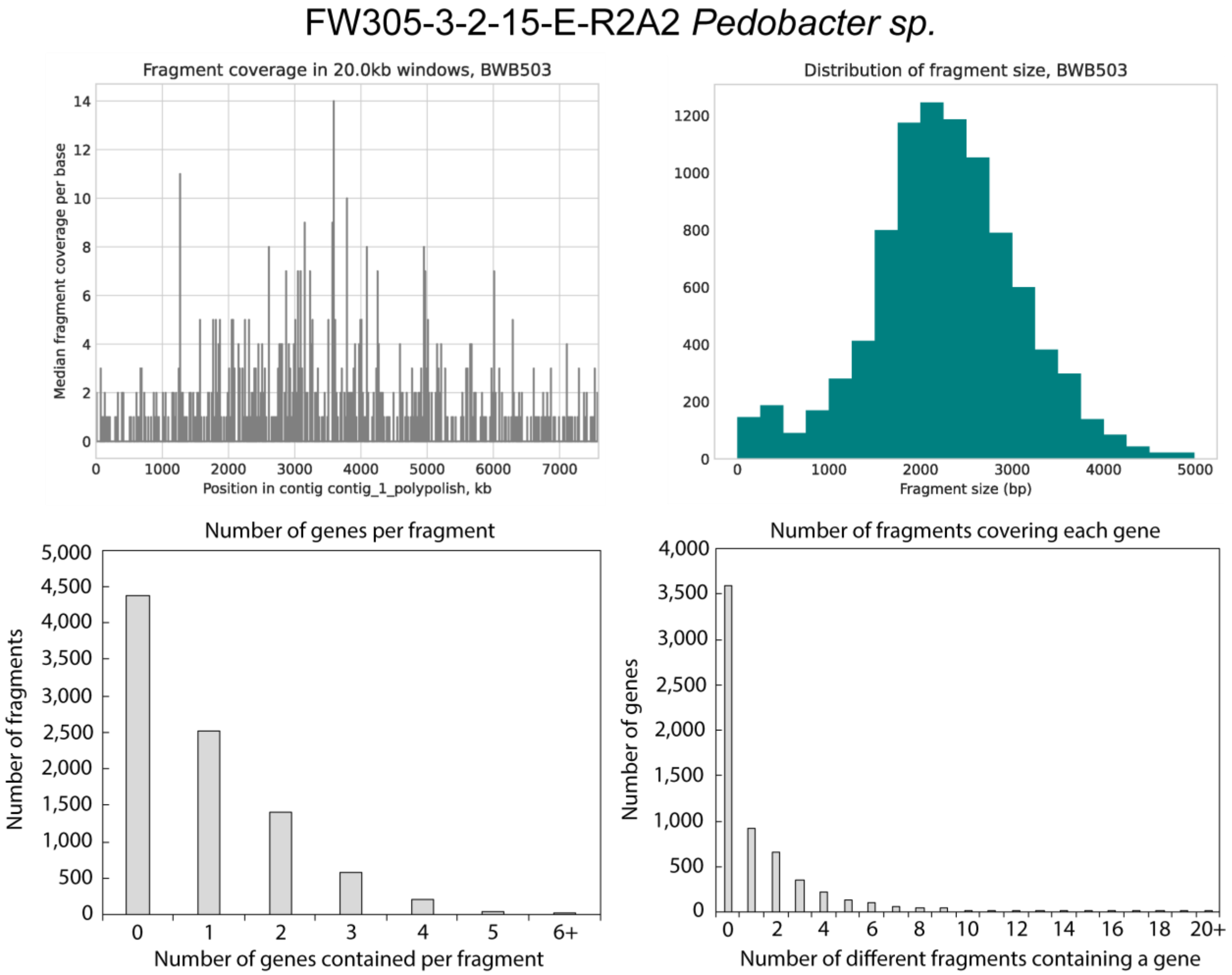

Appendix Figure S10. *FW305-3-2-15-E-R2A2 Pedobacter sp. fragment library quality figures.*

FHTAMBA *Acidovorax sp.*

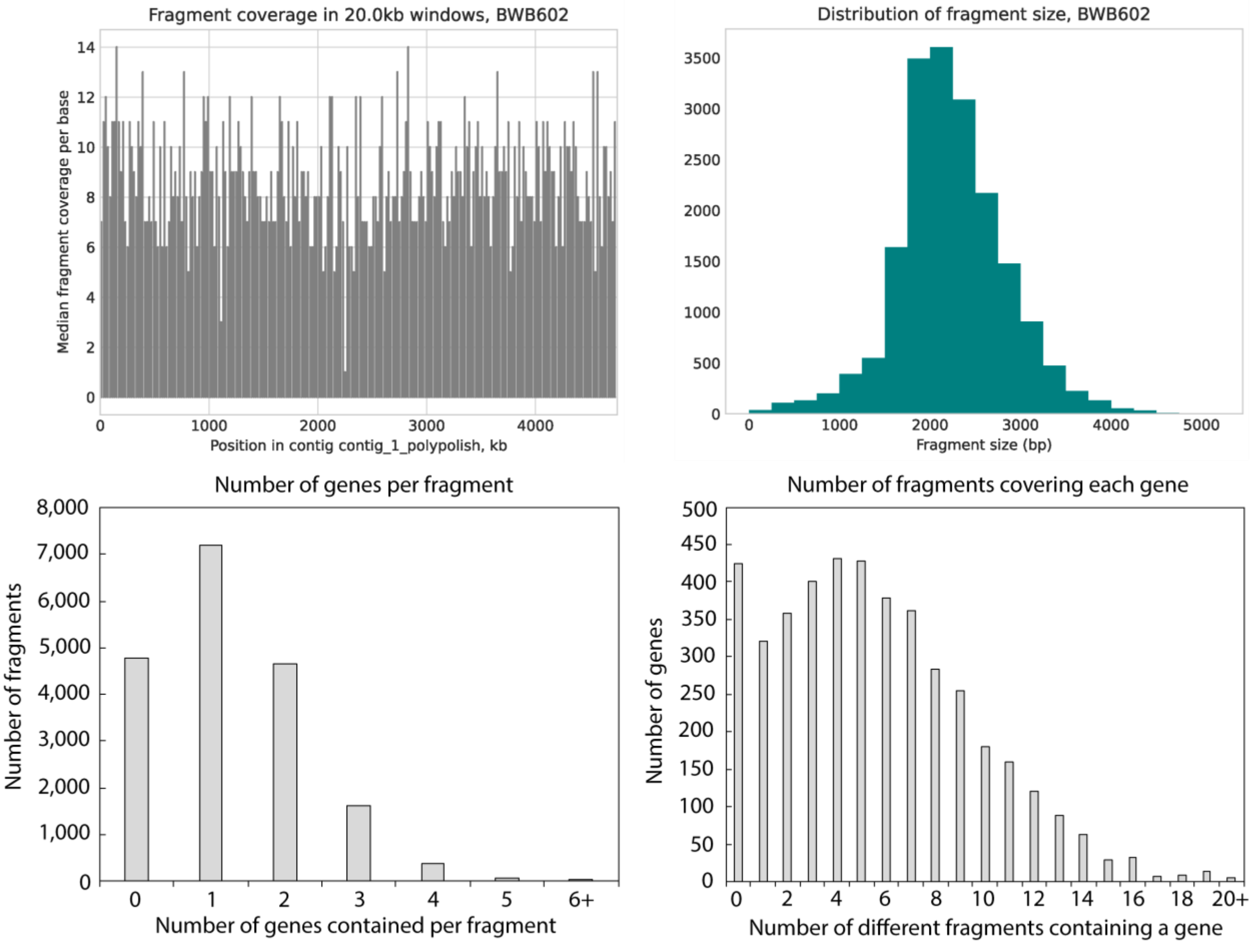

Appendix Figure S11. *FHTAMBA Acidovorax sp. Fragment library quality figures.*

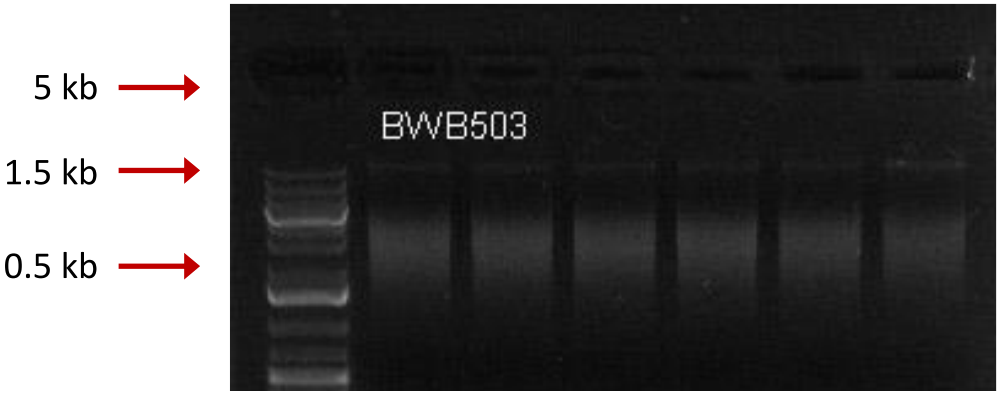

Appendix Figure S12. *Genome shearing example.* Example 1% DNA gel for Covaris ~3 kb shearing of 2 µg of genomic DNA from *Acidovorax sp*. FHTAMBA. Ladder shown is Thermo GeneRuler 1 kb plus. As can be seen, the smear centers around 3 kb, but spans from ~1-5 kb. When performing a gel extraction, the full range of the brightest portion of the band was excised.

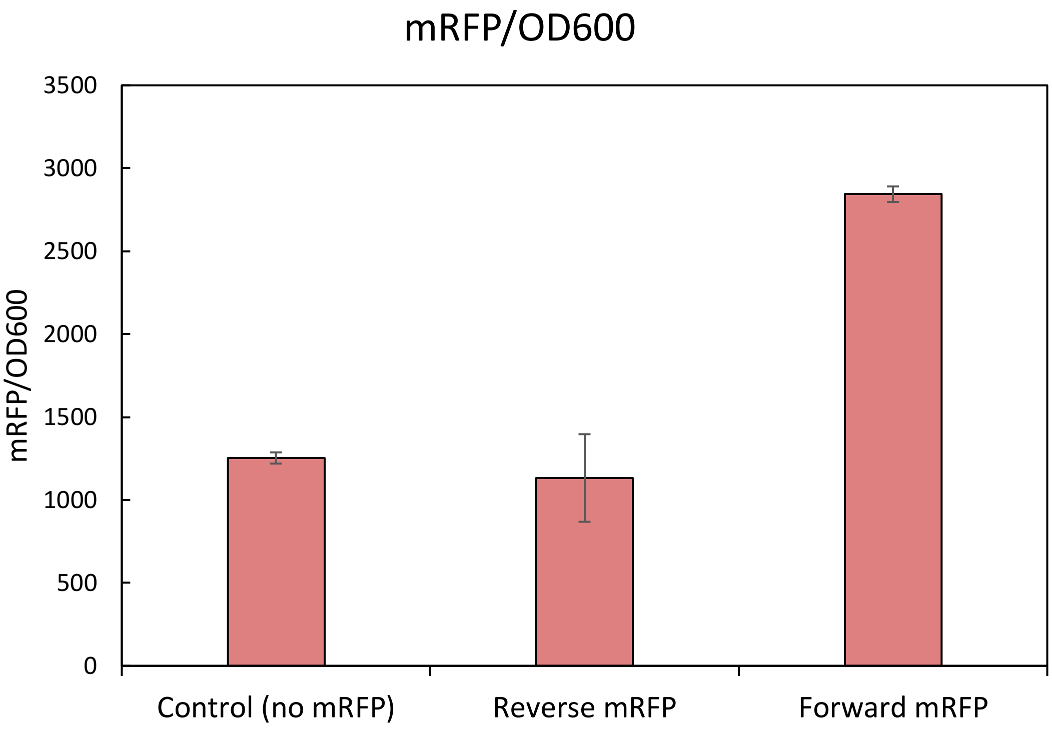

Appdendix Figure S13. *mRFP expression confirmation.* Three constructs tested to confirm expression from the synthetic promoter in the context of the vector used in this study. All constructs contain a Tet promoter and were induced with 1x aTc (100 ng/mL). The control lacked any mRFP gene. "Reverse mRFP" had the mRFP gene in the reverse orientation with respect to the synthetic promoter. "Forward mRFP" possessed the mRFP gene in the correct orientation with the synthetic promoter. Fluorescence was read with excitation at 587 nm and emission at 610 nm. Emission values were divided by the OD_{600} reading of the well. Values are for biological triplicate with the error bars representing standard deviation of biological triplicate.

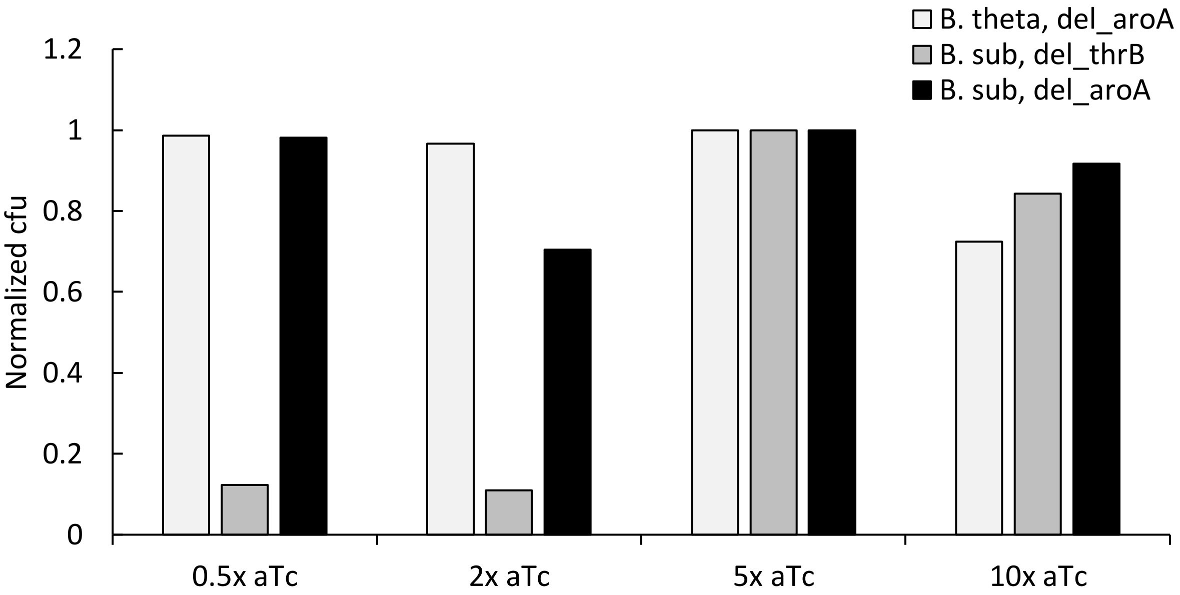

Appendix Figure S14. *Normalized colony forming units per aTc induction level for three complementation assays*. This test was specifically done with three different transformations of an individual genome fragment library into an auxotrophic strain. The lightest gray bar is for the transformation of the *B. thetaiotaomicron* library into ΔaroA. The medium shade gray bar is for the transformation of the *B. subtilis* library into ΔthrB. The black bar is for the transformation of the *B. subtilis* library into ΔaroA. Thus, we tested two different libraries and two different genetic knockout backgrounds to determine the optimum.

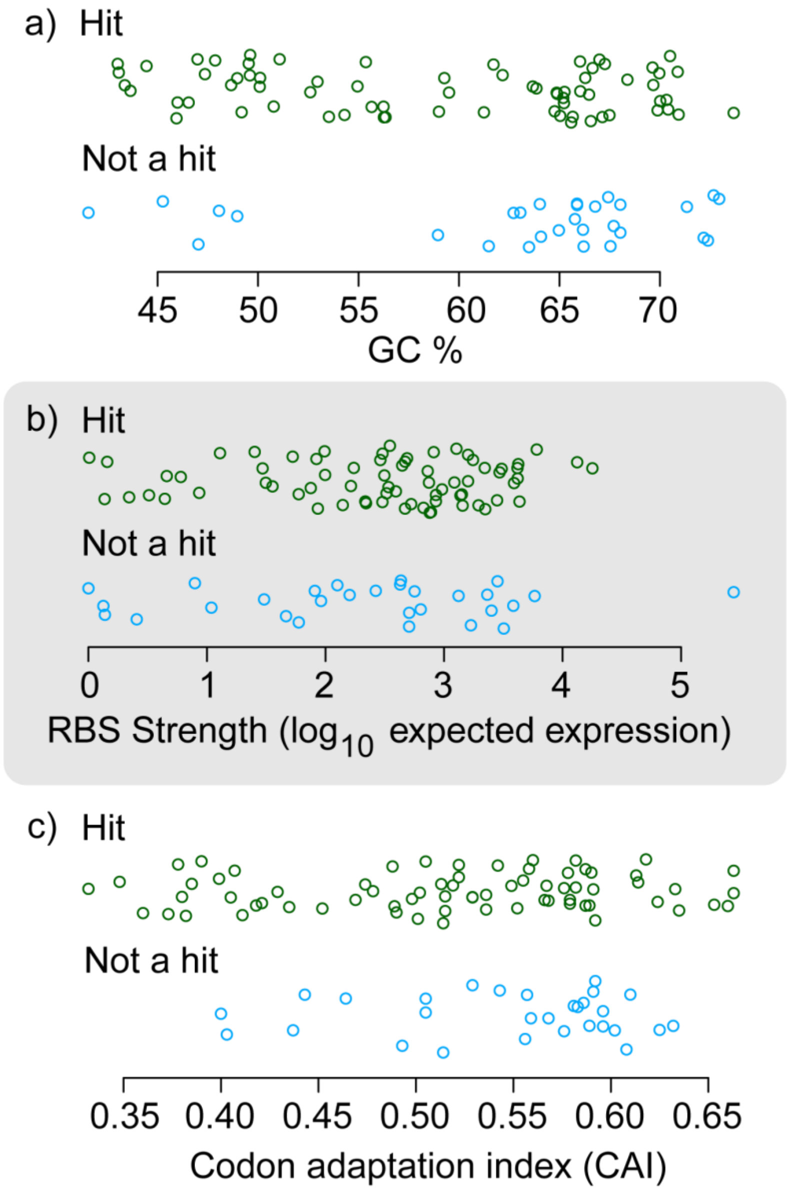

Appendix Figure S15. *Relationship between expected hit success and potential expression criteria.* This plot considers only expected hits that have at least one insert that "should" work (same strand and detected in the t_0). Among these, 69/ 99 were hits. As can be seen, when considering GC contents, predicted RBS strength, and codon adaptation index, there is no obvious trend to explain whether a gene would be successful, as successful and unsuccessful genes occupy almost entirely overlapping regions.

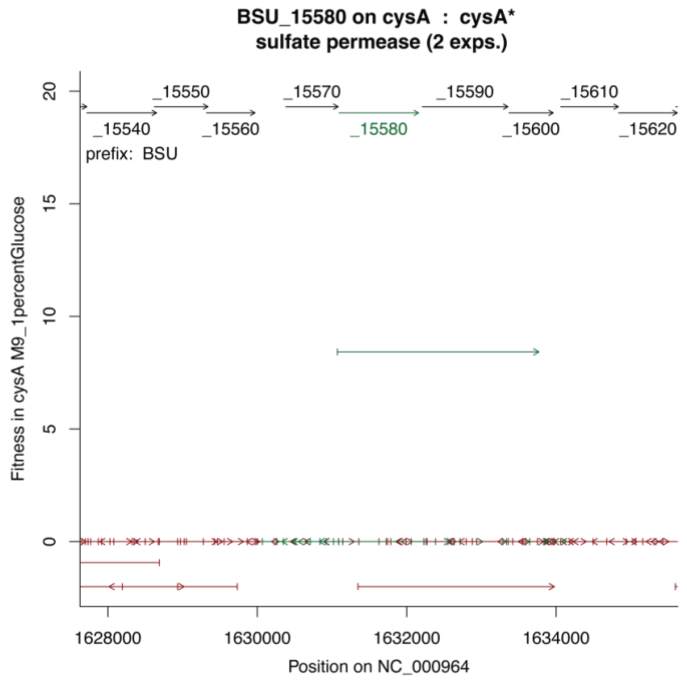

Appendix Figure S16. *Fitness benefit of BSU_15580 of B. subtilis associated fragments in the context of ΔcysA*. For Figures S16-S29, fragments that contain all of the gene of interest are highlighted in green. Fitness values (y-axis, log_2 fold-change) represent the average over two experiments. If a fragment is higher on the y-axis, it showed a greater fitness improvement. The top of the plot shows a reference segment of the source genome. Length of the fragments below gives an indication of which genes were included in the given fragment. The arrow on the fragment indicates its directionality within the expression vector.

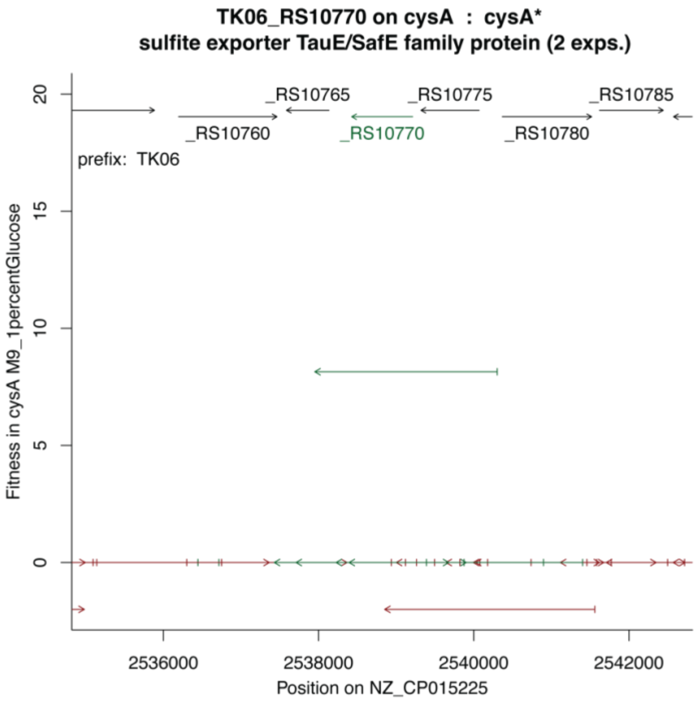

Appendix Figure S17. *Fitness benefit of TK06_RS10770 of P. fluorescens FW300-N2E2 associated fragments in the context of ΔcysA*.

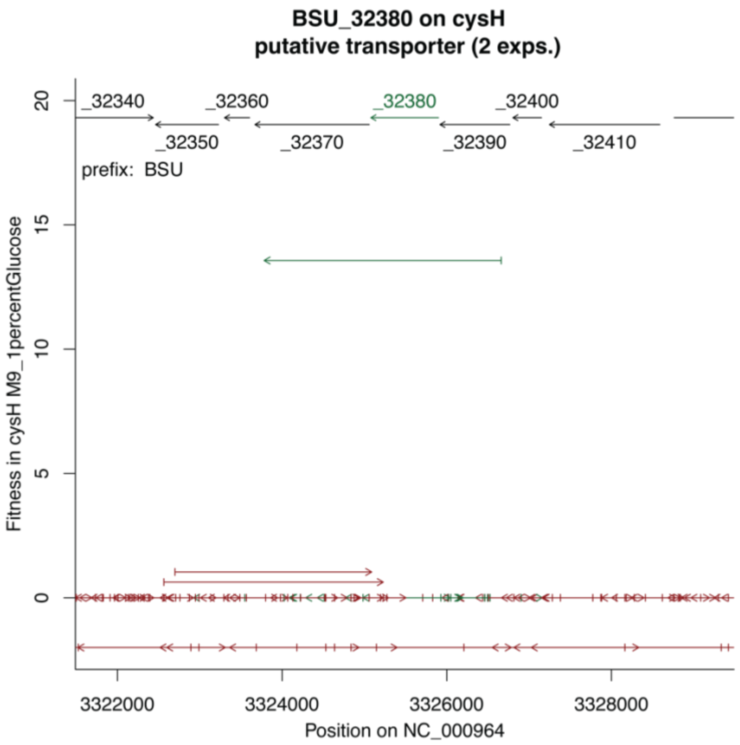

Appendix Figure S18. *Fitness benefit of B. subtilis BSU_32380 associated fragments in the context of ΔcysH.*

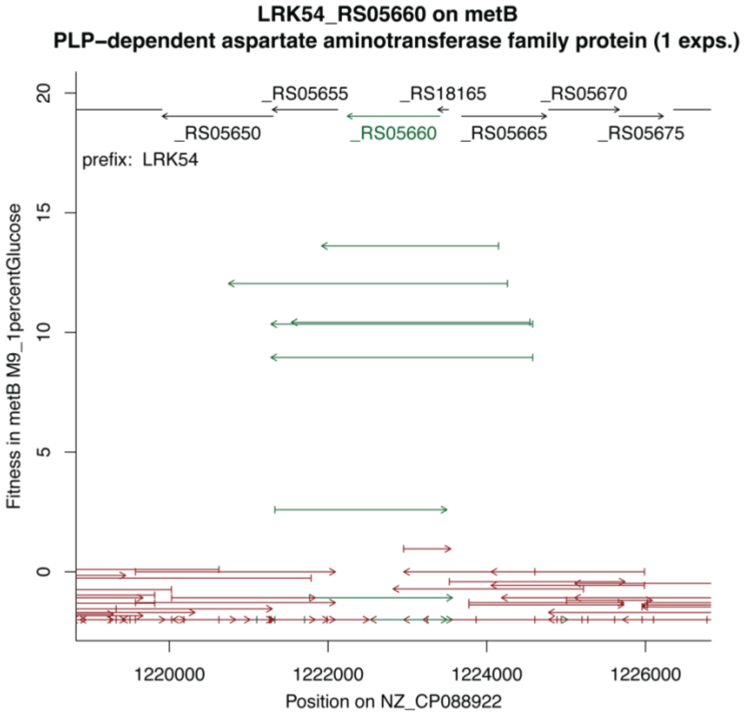

Appendix Figure S19. *Fitness benefit of LRK54_RS05660 of Rhodanobacter denitrificans FW104-10B01 associated fragments in the context of ΔmetB.*

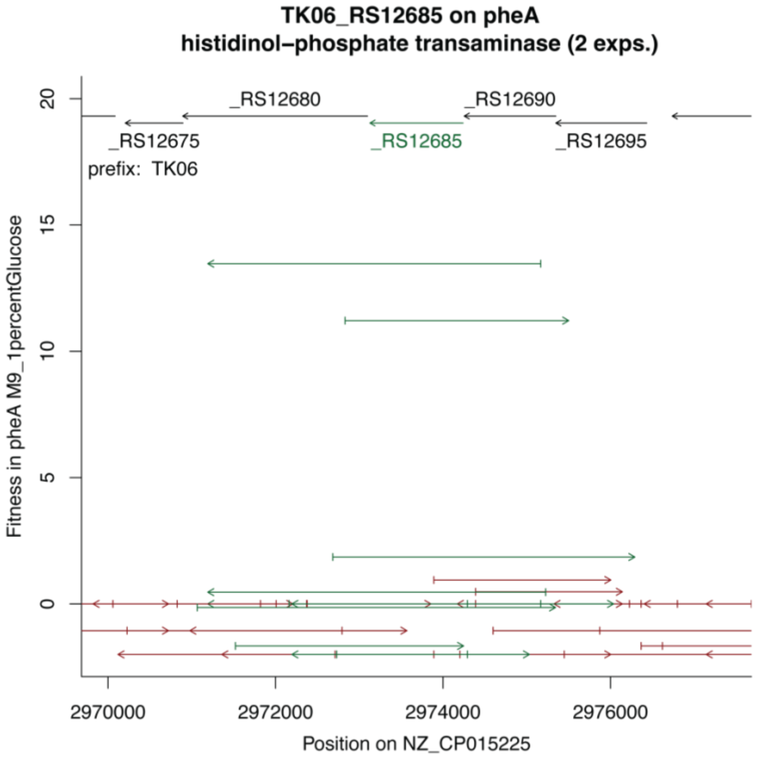

Appendix Figure S20. *Fitness benefit of TK06_RS12685 from P. fluorescens FW300-N2E2 associated fragments in the context of ΔpheA*.

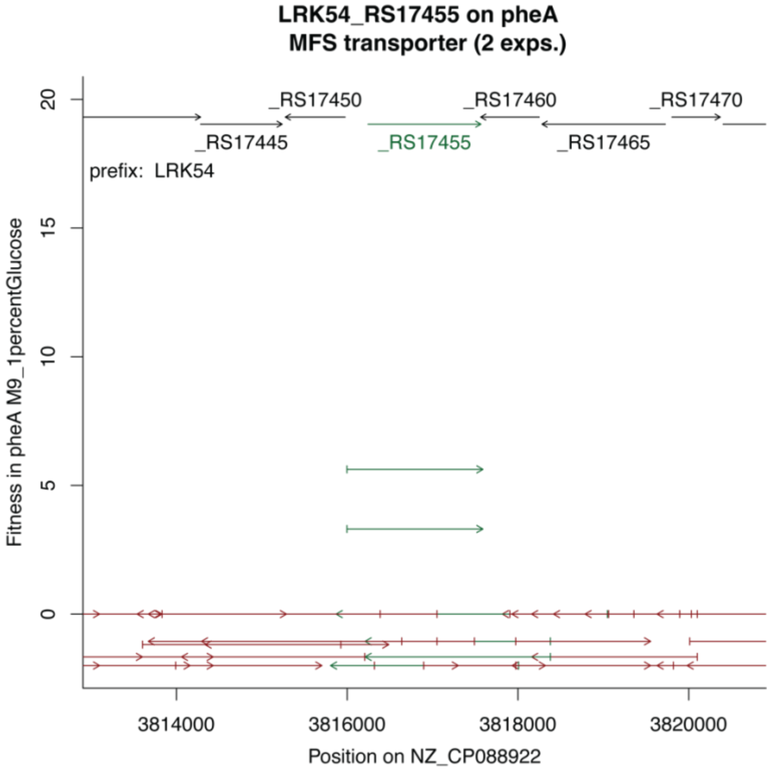

Appendix Figure S21. *Fitness benefit of LRK54_RS17455 of Rhodanobacter denitrificans FW104-10B01 associated fragments in the context of ΔpheA.*

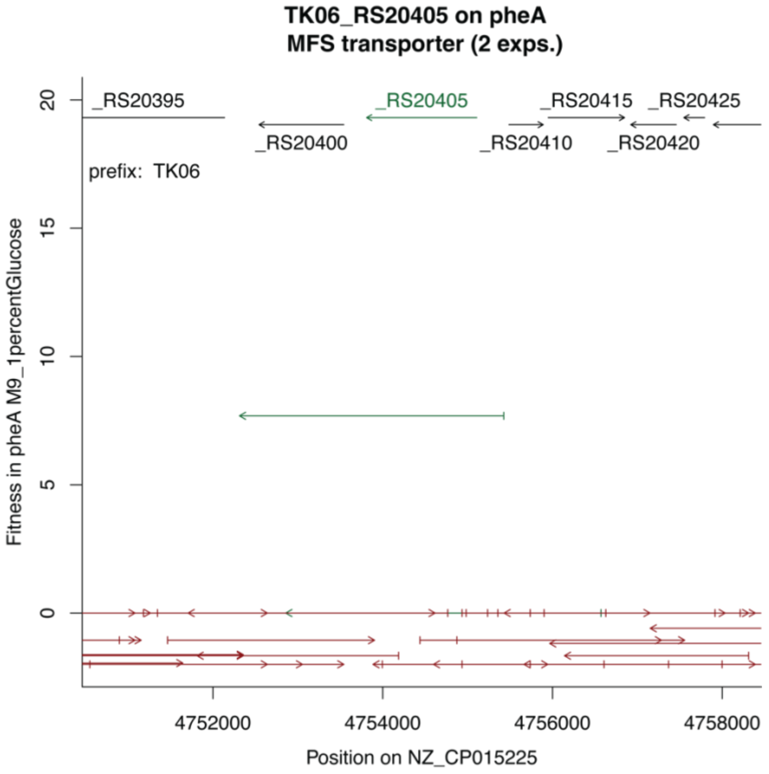

Appendix Figure S22. *Fitness benefit of TK06_RS20405 of P. fluorescens FW300-N2E2 associated fragments in the context of ΔpheA.*

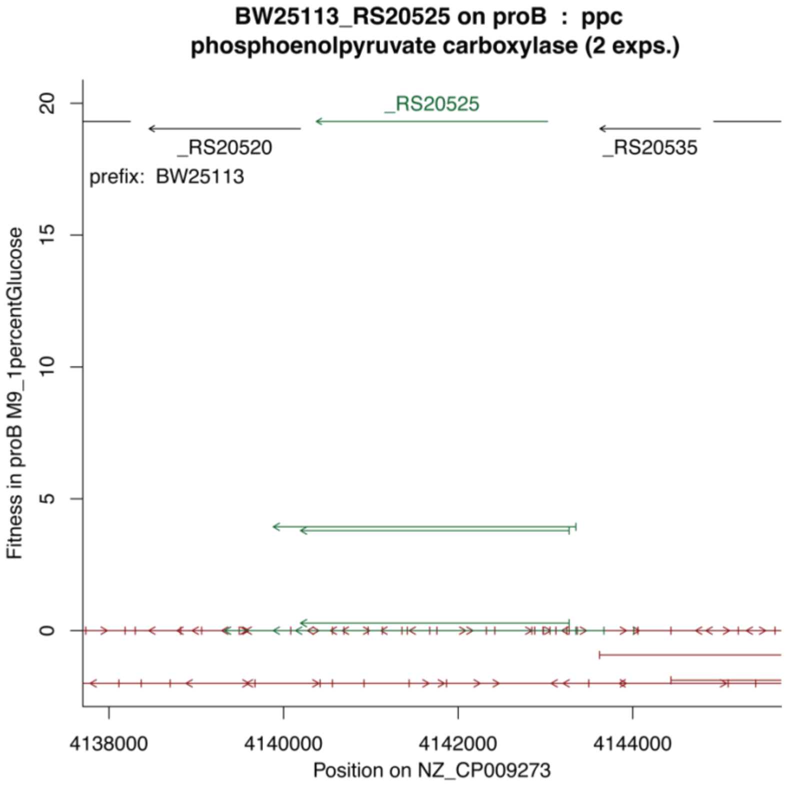

Appendix Figure S23. *Fitness benefit of E. coli BW25113_RS20525 associated fragments in the context of ΔproB.*

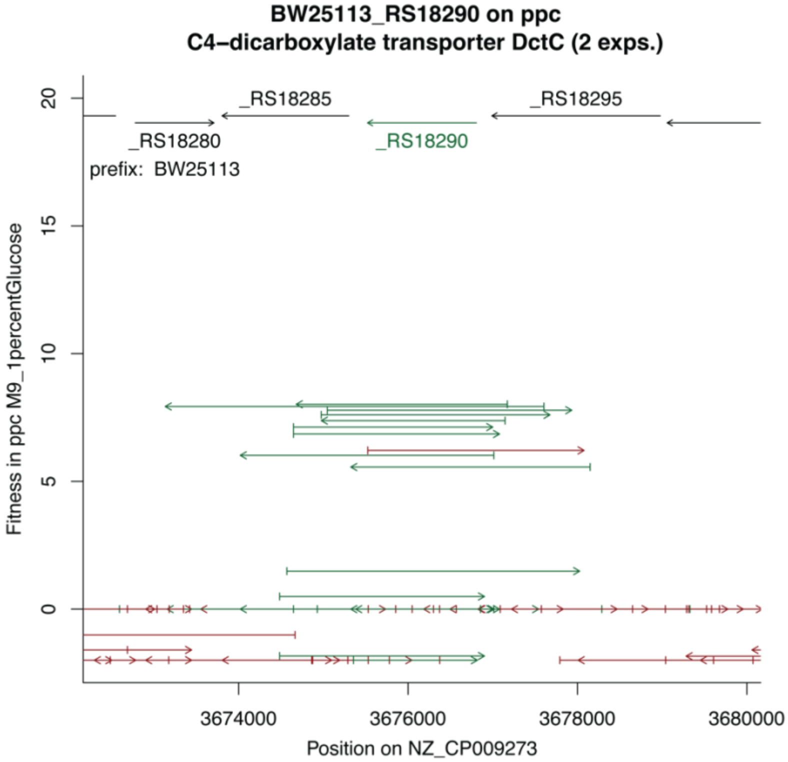

Appendix Figure S24. *Fitness benefit of E. coli BW25113_RS18290 associated fragments in the context of Δppc.*

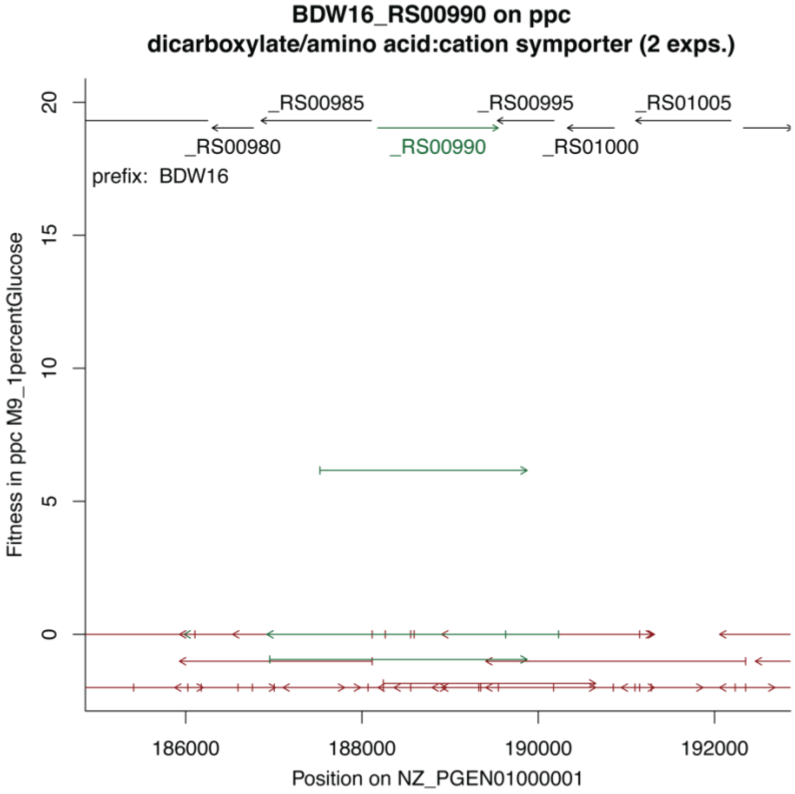

Appendix Figure S25. *Fitness benefit of BDW16_RS00990 of S. koreensis associated fragments in the context of Δppc.*

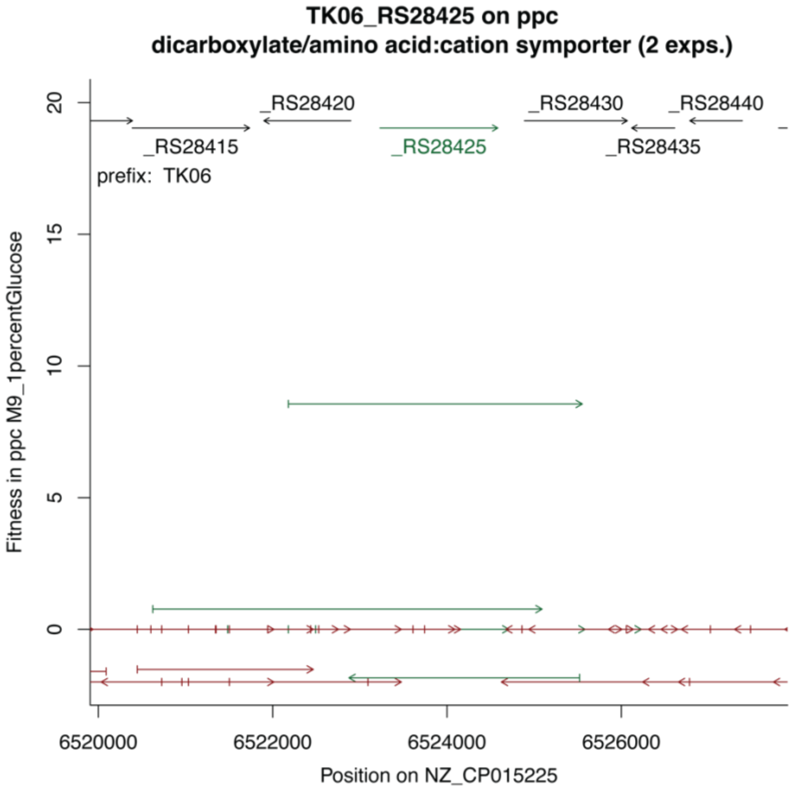

Appendix Figure S26. *Fitness benefit of TK06_RS28425 of P. fluorescens FW300-N2E2 associated fragments in the context of Δppc*.

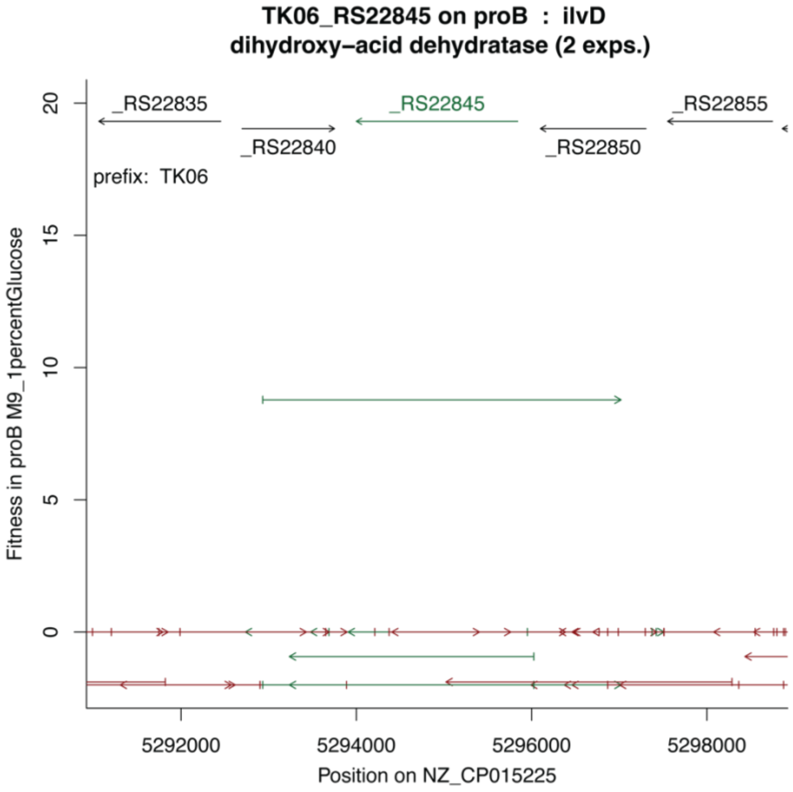

Appendix Figure S27. *Fitness benefit of TK06_RS22845 of P. fluorescens FW300-N2E2 associated fragments in the context of ΔproB.*

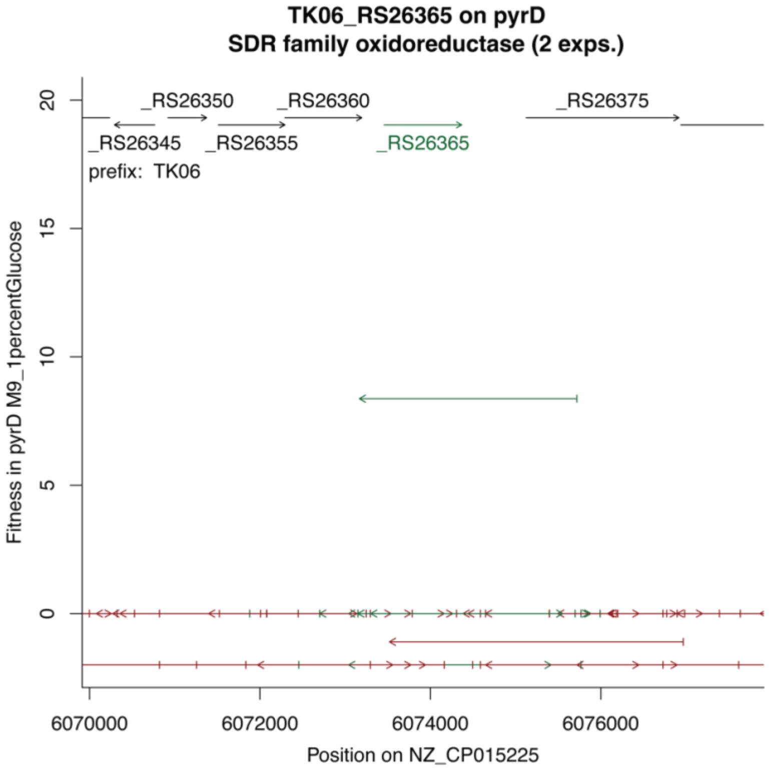

Appendix Figure S28. *Fitness benefit of TK06_RS26365 of P. fluorescens FW300-N2E2 associated fragments in the context of ΔpyrD.*

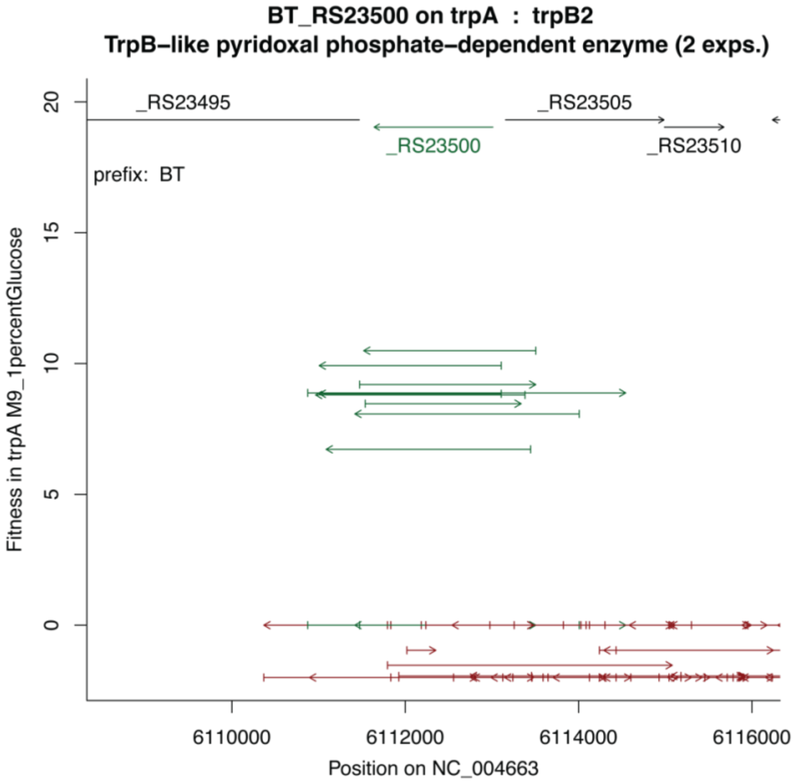

Appendix Figure S29. *Fitness benefit of BT_RS23500 of B. thetaiotaomicron associated fragments in the context of ΔtrpA.*

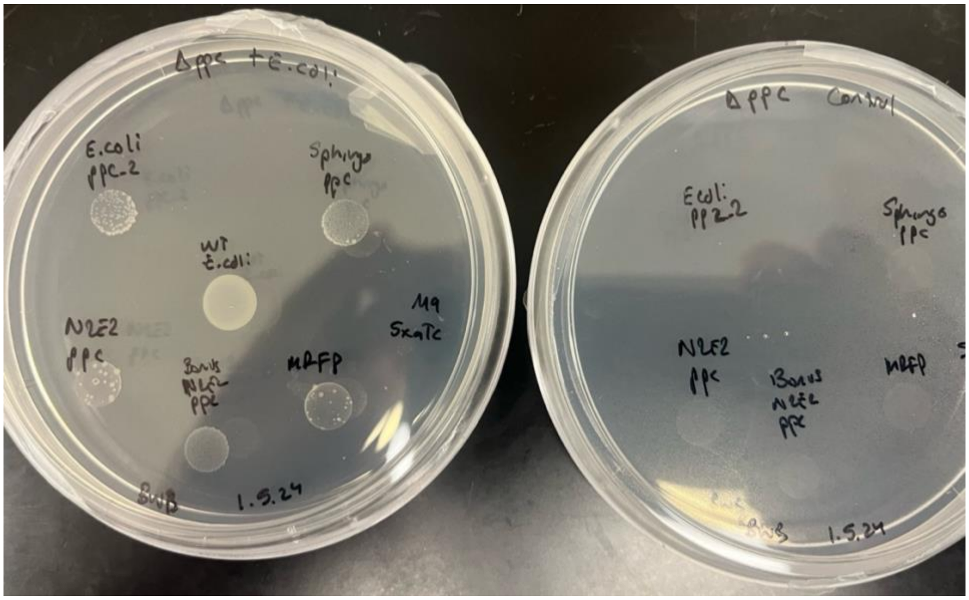

Appendix Figure S30. *Cross feeding with Δppc fitness enhancing fragments (dicarboxylic acid transporters)*. In the image are spotting assays of high-confidence hits (dicarboxylic acid transporters) identified in the context of Δ*ppc* rescue, spotted with (plate on the left) and without (plate on the right) wild-type *E. coli* on the same plate (spotted in the center). Candidate enzymes include BW25113_RS18290 of *E. coli* ("E. coli ppc_2", 10 o'clock), BDW16_RS00990 of *S. koreensis* ("Sphingo ppc", 2 o'clock), TK06_RS28425 of *P. fluorescens* FW300-N2E2 ("N2E2 ppc", 8 o'clock), along with a repeat of this condition that had better transformation efficiency ("Bonus N2E2 ppc", 6 o'clock). The control ("mRFP") can be found at 4 o'clock. As can be seen, there is not only a leading edge towards the wild-type *E. coli*, but the control (mRFP) lacks similar growth. This suggests that the wild-type *E. coli* is secreting the required nutrient to allow for grow of this knockout strain. And while it is possible for the control to take up this nutrient (there are some colonies on the mRFP control), there is a significant benefit to overexpression of the transporters (additional growth, leading edge). None of the strains grow without the wild-type *E. coli* spotted (plate on the right). The cutoff portion of the right plate reads M9 5x aTc (description of plate). There was no cell growth out of frame.

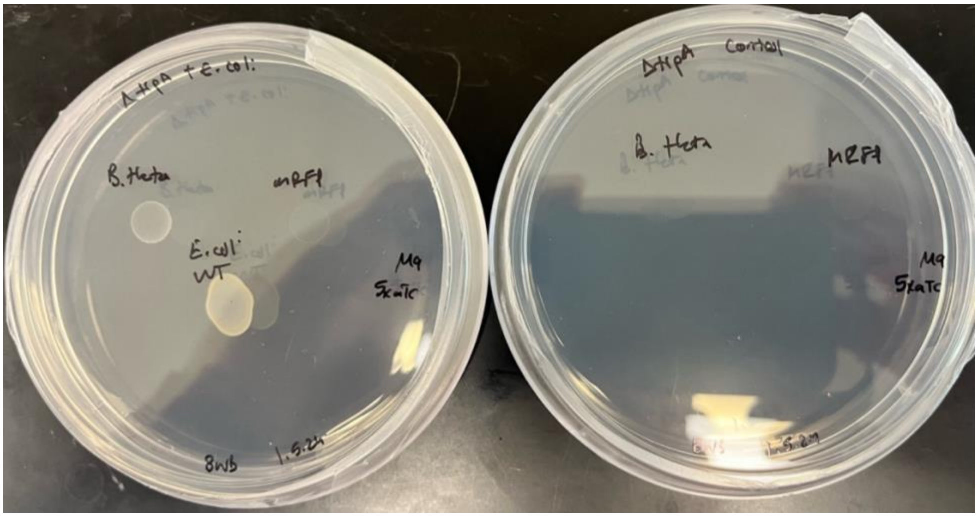

Appendix Figure S31. *Cross feeding in the context of the ΔtrpA fitness enhancing fragment.* Similar to the ppc high-confidence candidates, it can be seen that spotting the trpA candidate with (left) and without (right) wild-type *E. coli* shows some level cross feeding that allows for rescued growth. For the plate on the left, the strain containing the trpB2 candidate BT_RS23500 of *B. thetaiotaomicron* ("B. theta", 10 o'clock) shows growth while the control ("mRFP", 2 o'clock) does not. Without *E. coli* spotted, plate on the right, neither strain grows. As the proposed function of the trpB2 gene is use of indole to synthesize tryptophan, it is likely that indole is being secreted by wild-type *E. coli* and taken up by the strain overexpressing the trpB2 candidate from *B. thetaiotaomicron*, as direct provision of tryptophan itself would likely allow for the control to grow as well.

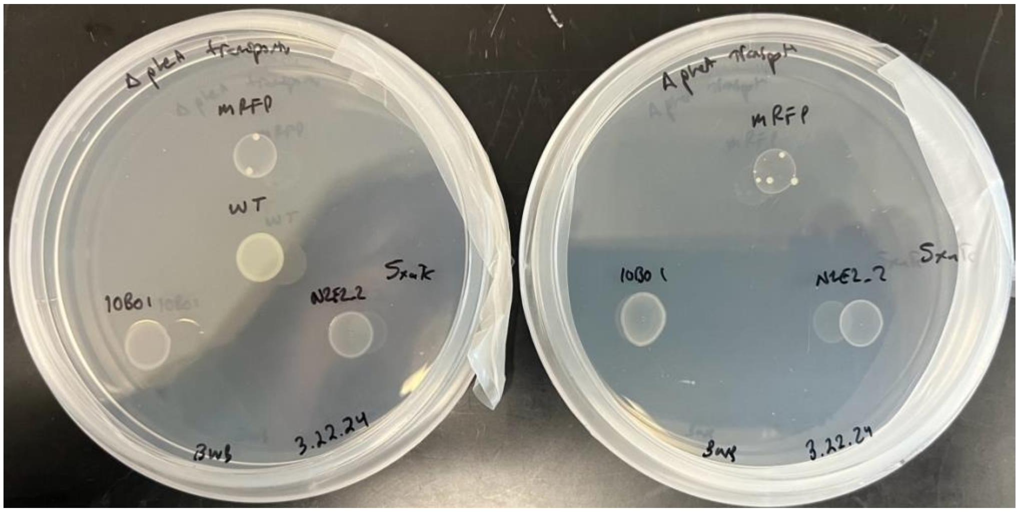

Appendix Figure S32. *Cross feeding in the context of ΔpheA fitness enhancing fragments containing putative MFS transporters LRK54_RS17455 and TK06_RS20405*. Spotting assays with LRK54_RS17455 of *Rhodanobacter denitrificans* FW104-10B01, TK06_RS20405 of *P. fluorescens* FW300-N2E2, and the mRFP control both with and without wild-type *E. coli* also spotted on the plate. Through the overall growth seems higher for the two MFS transporter candidate overexpressing strains compared to the mRFP control, the pattern is not differentiable between the cases where *E. coli* has and has not been co-spotted. Thus, it is not possible to confirmed a benefit of cross feeding.

Supplemental Tables

Knockout	Pathway	Function
ΔaroA	chorismate biosynthesis (amino acid)	3-phosphoshikimate 1-carboxyvinyltransferase
ΔthrB	L-threonine biosynthesis (amino acid)	Homoserine kinase
ΔmetB	L-methionine biosynthesis (amnio acid)	Cystathionine gamma-synthase
ΔproB	L-proline biosynthesis (amino acid)	Glutamate 5-kinase
ΔproA	L-proline biosynthesis (amino acid)	Gamma-glutamyl phosphate reductase
ΔcysA	sulfate transport (aminio acid)	Sulfate/thiosulfate import ATP-binding protein CysA
ΔargG	L-arginine biosynthesis (amino acid)	Argininosuccinate synthase
ΔleuA	L-leucine biosynthesis (amino acid)	2-isopropylmalate synthase
ΔtrpA	L-tryptophan biosynthesis (amino acid)	Tryptophan synthase alpha chain
ΔserA	L-serine biosynthesis (amino acid)	D-3-phosphoglycerate dehydrogenase
ΔhisC	L-histidine biosynthesis (amino acid)	Histidinol-phosphate aminotransferase
ΔmetE	L-methionine biosynthesis (amnio acid)	5-methyltetrahydropteroyltriglutamate--homocysteine methyltransferase
ΔilvD	valine/isoleucine biosynthesis (amino acid)	Dihydroxy-acid dehydratase
ΔpheA	tyrosine/phenylalanine biosynthesis (amino acid)	Bifunctional chorismate mutase/prephenate dehydratase
ΔpurE	inosine-5'-phosphate biosynthesis (nucleotide)	N5-carboxyaminoimidazole ribonucleotide mutase
ΔcysH	assimilatory sulfate reduction (amino acid)	Phosphoadenosine 5'-phosphosulfate reductase
ΔhisG	L-histidine biosynthesis (amino acid)	ATP phosphoribosyltransferase
ΔaroE	chorismate biosynthesis (amino acid)	Shikimate dehydrogenase (NADP(+))
ΔpyrD	UMP biosynthesis (nucleotide)	Dihydroorotate dehydrogenase (quinone)
Δppc	Mixed acid fermentation	Phosphoenolpyruvate carboxylase

Appendix Table S1. *Table of E. coli knockout auxotrophic strain backgrounds and missing function.* The first column gives the knockout. The second column shows biochemical step, and where relevant, the larger pathway it is part of. The third and final column shows the biochemical activity of the protein.

Name	Fragment Library	KO	Transformation Efficiency (cfus)
BBL1	pBWBHTC5, *Bacteroides thetaiotaomicron*	ΔthrB	170,000
BBL2	pBWBHTC5, *Bacteroides thetaiotaomicron*	ΔaroA	73,000
BBL3	pBWBHTC4, *Bacillus subtilis* 168	ΔthrB	178,000
BBL4	pBWBHTC4, *Bacillus subtilis* 168	ΔaroA	80,000
BBL5	pBWBHTC5, *Bacteroides thetaiotaomicron*	ΔmetB	9750000
BBL6	pBWBHTC5, *Bacteroides thetaiotaomicron*	ΔproA	14300000
BBL7	pBWBHTC4, *Bacillus subtilis* 168	ΔmetB	40300000
BBL8	pBWBHTC4, *Bacillus subtilis* 168	ΔproA	7150000
BBL9	pBWBHTC1, *Escherichia coli* BW25113	ΔmetB	1592500

BBL10	pBWBHTC1, *Escherichia coli* BW25113	ΔproA	5200000
BBL11	pBWBHTC2, *Pseudomonas fluorescens* (FW300-N2E2)	ΔmetB	1560000
BBL12	pBWBHTC2, *Pseudomonas fluorescens* (FW300-N2E2)	ΔproA	975000
BBL13	pBWBHTC3, *Sphingomonas koreensis*	ΔproA	3900000
BBL14	pBWBHTC6, BWB514, FW306-1B-D06B, *Lysobacter sp*.	ΔproA	5200000
BBL15	pBWBHTC7, BWB549, GW821-FHT01B05, *Xylophilus sp*.	ΔproA	3445000
BBL16	pBWBHTC8, BWB510, FW104-10B01, *Rhodanobacter denitrificans*	ΔproA	3683333
BBL17	pBWBHTC9 - BWB553 GW822-FHT02A01, *Rhodoferax sp*.	ΔproA	26000
BBL18	pBWBHTC10 - BWB503, FW305-3-2-15-E-R2A2, *Pedobacter sp*.	ΔproA	1300000
BBL19	pBWBHTC11 - BWB602, FHTAMBA, *Acidovorax sp*.	ΔproA	6500
BBL20	pBWBHTC3 - *Sphingomonas koreensis*	ΔmetB	17550000
BBL21	pBWBHTC6, BWB514, FW306-1B-D06B, *Lysobacter sp*.	ΔmetB	5200000
BBL22	pBWBHTC7, BWB549, GW821-FHT01B05, *Xylophilus sp*.	ΔmetB	2600000
BBL23	pBWBHTC8, BWB510, FW104-10B01, *Rhodanobacter denitrificans*	ΔmetB	5850000
BBL24	pBWBHTC9 - BWB553 GW822-FHT02A01, *Rhodoferax sp*.	ΔmetB	130000
BBL25	pBWBHTC10 - BWB503, FW305-3-2-15-E-R2A2, *Pedobacter sp*.	ΔmetB	19500000
BBL26	pBWBHTC11 - BWB602, FHTAMBA, *Acidovorax sp*.	ΔmetB	4875000
BBL27	pBWBHTC1, *Escherichia coli* BW25113	ΔaroA	1170000
BBL28	pBWBHTC2, *Pseudomonas fluorescens* (FW300-N2E2)	ΔaroA	4268333.333
BBL29	pBWBHTC3, *Sphingomonas koreensis*	ΔaroA	17225000
BBL30	pBWBHTC6, BWB514, FW306-1B-D06B, *Lysobacter sp*.	ΔaroA	2600000
BBL31	pBWBHTC7, BWB549, GW821-FHT01B05, *Xylophilus sp*.	ΔaroA	10075000
BBL32	pBWBHTC8, BWB510, FW104-10B01, *Rhodanobacter denitrificans*	ΔaroA	3250000
BBL33	pBWBHTC9 - BWB553 GW822-FHT02A01, *Rhodoferax sp*.	ΔaroA	39000
BBL34	pBWBHTC10 - BWB503, FW305-3-2-15-E-R2A2, *Pedobacter sp*.	ΔaroA	17550000
BBL35	pBWBHTC11 - BWB602, FHTAMBA, *Acidovorax sp.*	ΔaroA	1495000
BBL36	pBWBHTC1, *Escherichia coli* BW25113	ΔthrB	585000
BBL37	pBWBHTC2, *Pseudomonas fluorescens* (FW300-N2E2)	ΔthrB	910000
BBL38	pBWBHTC3, *Sphingomonas koreensis*	ΔthrB	1657500
BBL39	pBWBHTC6, BWB514, FW306-1B-D06B, *Lysobacter sp.*	ΔthrB	910000
BBL40	pBWBHTC7, BWB549, GW821-FHT01B05, *Xylophilus sp.*	ΔthrB	1137500
BBL41	pBWBHTC8, BWB510, FW104-10B01, *Rhodanobacter denitrificans*	ΔthrB	1657500
BBL42	pBWBHTC9 - BWB553 GW822-FHT02A01, *Rhodoferax sp.*	ΔthrB	19500

BBL43	pBWBHTC10 - BWB503, FW305-3-2-15-E-R2A2, *Pedobacter sp.*	ΔthrB	6500000
BBL44	pBWBHTC11 - BWB602, FHTAMBA, *Acidovorax sp.*	ΔthrB	2632500
BBL45	Mix of all 11	ΔthrB	910000
BBL46	Mix of all 11	ΔaroA	81250
BBL47	Mix of all 11	ΔmetB	1235000
BBL48	Mix of all 11	ΔproA	780000
BBL49	Mix of all 11	ΔCysA	113750
BBL50	Mix of all 11	ΔArgG	6175000
BBL51	Mix of all 11	ΔLeuA	487500
BBL52	Mix of all 11	ΔtrpA	877500
BBL53	Mix of all 11	ΔserA	975000
BBL54	Mix of all 11	ΔhisC	5200000
BBL55	Mix of all 11	ΔpheA	16900000
BBL56	Mix of all 11	ΔpurE	2827500
BBL57	Mix of all 11	Δppc	1170000
BBL58	Mix of all 11	ΔproB	650000
BBL59	Mix of all 11	ΔcysH	325000
BBL60	Mix of all 11	ΔmetE	5525000
BBL61	Mix of all 11	ΔserB	2925000
BBL62	Mix of all 11	ΔhisG	1040000
BBL63	Mix of all 11	ΔaroE	2210000
BBL64	Mix of all 11	ΔpyrD	1072500
BBL65	Mix of all 11	ΔilvD	1690000

Appendix Table S2. Summary of all transformations run. The first column gives the shorthand for the transformation (BBL###). These are the t_0 samples. The second column describes the genomic libraries that were transformed in. The third column gives the genetic knockout of the auxotrophic strain. The final column give the transformation efficiency in colony forming units (spotting).

Name	Fragment Library	Reference Transformation	KO destination	aTc concentration
BBS1	pBWBHTC5 – *Bacteroides thetaiotaomicron*	BBL2	ΔaroA	5x
BBS2	pBWBHTC4, *Bacillus subtilis* 168	BBL3	ΔthrB	5x
BBS3	pBWBHTC4, *Bacillus subtilis* 168	BBL4	ΔaroA	5x
BBS4	pBWBHTC5 – *Bacteroides thetaiotaomicron*	BBL2	ΔaroA	0.5x
BBS5	pBWBHTC5 – *Bacteroides thetaiotaomicron*	BBL2	ΔaroA	10x
BBS6	pBWBHTC4, *Bacillus subtilis* 168	BBL3	ΔthrB	0.5x
BBS7	pBWBHTC4, *Bacillus subtilis* 168	BBL3	ΔthrB	10x
BBS8	pBWBHTC4, *Bacillus subtilis* 168	BBL4	ΔaroA	0.5x
BBS9	pBWBHTC4, *Bacillus subtilis* 168	BBL4	ΔaroA	10x

BBS10	pBWBHTC4, *Bacillus subtilis* 168	BBL7	ΔmetB	1x
BBS11	pBWBHTC4, *Bacillus subtilis* 168	BBL7	ΔmetB	5x
BBS12	pBWBHTC4, *Bacillus subtilis* 168	BBL7	ΔmetB	10x
BBS13	pBWBHTC4, *Bacillus subtilis* 168	BBL8	ΔproA	1x
BBS14	pBWBHTC4, *Bacillus subtilis* 168	BBL8	ΔproA	10x
BBS15	pBWBHTC1, *Escherichia coli* BW25113	BBL10	ΔproA	1x
BBS16	pBWBHTC1, *Escherichia coli* BW25113	BBL9	ΔmetB	1x
BBS17	pBWBHTC1, *Escherichia coli* BW25113	BBL9	ΔmetB	5x
BBS18	pBWBHTC8, BWB510, FW104-10B01, *Rhodanobacter denitrificans*	BBL16	ΔproA	1x
BBS19	pBWBHTC8, BWB510, FW104-10B01, *Rhodanobacter denitrificans*	BBL16	ΔproA	5x
BBS20	pBWBHTC7, BWB549, GW821-FHT01B05, *Xylophilus sp*.	BBL15	ΔproA	1x, 5x
BBS21	pBWBHTC2, *Pseudomonas fluorescens* (FW300-N2E2)	BBL12	ΔproA	1x, 5x
BBS22	pBWBHTC6 – BWB514, FW306-1B-D06B, *Lysobacter sp*.	BBL14	ΔproA	1x, 5x
BBS23	pBWBHTC5 – *Bacteroides thetaiotaomicron*	BBL5	ΔmetB	1x
BBS24	pBWBHTC5 – *Bacteroides thetaiotaomicron*	BBL6	ΔproA	1x, 5x, 10x
BBS25	pBWBHTC3, *Sphingomonas koreensis*	BBL13	ΔproA	5x
BBS26	pBWBHTC11 – BWB602, FHTAMBA, *Acidovorax sp*.	BBL26	ΔmetB	1x, 5x
BBS27	pBWBHTC9 – BWB553 GW822-FHT02A01, *Rhodoferax sp*.	BBL24	ΔmetB	1x, 5x
BBS28	pBWBHTC10 – BWB503, FW305-3-2-15-E-R2A2, *Pedobacter sp*.	BBL18	ΔproA	1x, 5x
BBS29	pBWBHTC11 – BWB602, FHTAMBA, *Acidovorax sp*.	BBL19	ΔproA	1x, 5x
BBS30	pBWBHTC9 – BWB553 GW822-FHT02A01, *Rhodoferax sp*.	BBL17	ΔproA	1x, 5x
BBS31	pBWBHTC6 – BWB514, FW306-1B-D06B, *Lysobacter sp*.	BBL21	ΔmetB	1x, 5x
BBS32	pBWBHTC1, *Escherichia coli* BW25113	BBL27	ΔaroA	1x, 5x
BBS33	pBWBHTC2, *Pseudomonas fluorescens* (FW300-N2E2)	BBL28	ΔaroA	1x, 5x
BBS34	pBWBHTC8, BWB510, FW104-10B01, *Rhodanobacter denitrificans*	BBL32	ΔaroA	1x, 5x
BBS35	pBWBHTC10 – BWB503, FW305-3-2-15-E-R2A2, *Pedobacter sp*.	BBL34	ΔaroA	1x, 5x
BBS36	pBWBHTC11 – BWB602, FHTAMBA, *Acidovorax sp*.	BBL35	ΔaroA	1x, 5x
BBS37	pBWBHTC8, BWB510, FW104-10B01, *Rhodanobacter denitrificans*	BBL23	ΔmetB	5x

BBS38	pBWBHTC1, Escherichia coli BW25113	BBL36	ΔthrB	5x
BBS39	pBWBHTC2, *Pseudomonas fluorescens* (FW300-N2E2)	BBL37	ΔthrB	5x
BBS40	pBWBHTC6 – BWB514, FW306-1B-D06B, *Lysobacter sp*.	BBL39	ΔthrB	1x, 5x
BBS41	pBWBHTC7, BWB549, GW821-FHT01B05, *Xylophilus sp*.	BBL40	ΔthrB	5x
BBS42	pBWBHTC3, *Sphingomonas koreensis*	BBL38	ΔthrB	1x, 5x
BBS43	Mix -11	BBL45	ΔthrB	1x
BBS44	Mix -11	BBL45	ΔthrB	5x
BBS45	Mix -11	BBL47	ΔmetB	1x
BBS46	Mix -11	BBL47	ΔmetB	5x
BBS47	pBWBHTC9 – BWB553 GW822-FHT02A01, *Rhodoferax sp*.	BBL33	ΔaroA	5x
BBS48	pBWBHTC8, BWB510, FW104-10B01, *Rhodanobacter denitrificans*	BBL41	ΔthrB	1x, 5x
BBS49	Mix – 11	BBL48	ΔproA	1x, 5x
BBS50	Mix – 11	BBL46	ΔaroA	1x
BBS51	pBWBHTC11 – BWB602, FHTAMBA, *Acidovorax sp*.	BBL44	ΔthrB	1x, 5x
BBS52	pBWBHTC9 – BWB553 GW822-FHT02A01, *Rhodoferax sp*.	BBL42	ΔthrB	1x, 5x
BBS53	Mix – 11	BBL46	ΔaroA	5x
BBS54	Mix – 11	BBL49	ΔcysA	1x
BBS55	Mix – 11	BBL49	ΔcysA	5x
BBS56	Mix – 11	BBL51	ΔleuA	1x
BBS57	Mix – 11	BBL51	ΔleuA	5x
BBS58	Mix – 11	BBL52	ΔtrpA	1x
BBS59	Mix – 11	BBL52	ΔtrpA	5x
BBS60	Mix – 11	BBL50	ΔargA	1x
BBS61	Mix – 11	BBL50	ΔargA	5x
BBS62	Mix – 11	BBL53	ΔserA	1x
BBS63	Mix – 11	BBL53	ΔserA	5x
BBS64	Mix – 11	BBL55	ΔpheC	1x
BBS65	Mix – 11	BBL55	ΔpheC	5x
BBS66	Mix – 11	BBL56	ΔpurE	1x
BBS67	Mix – 11	BBL56	ΔpurE	5x
BBS68	Mix – 11	BBL54	ΔhisC	1x
BBS69	Mix – 11	BBL54	ΔhisC	5x
BBS70	Mix – 11	BBL57	Δppc	1x
BBS71	Mix – 11	BBL57	Δppc	5x

BBS72	Mix – 11	BBL58	ΔproB	1x
BBS73	Mix – 11	BBL58	ΔproB	5x
BBS74	Mix – 11	BBL60	ΔmetE	1x
BBS75	Mix – 11	BBL60	ΔmetE	5x
BBS76	Mix – 11	BBL61	ΔserB	1x
BBS77	Mix – 11	BBL61	ΔserB	5x
BBS78	Mix – 11	BBL63	ΔaroE	1x
BBS79	Mix – 11	BBL63	ΔaroE	5x
BBS80	Mix – 11	BBL64	ΔpyrD	1x
BBS81	Mix – 11	BBL64	ΔpyrD	5x
BBS82	Mix – 11	BBL65	ΔilvD	1x
BBS83	Mix – 11	BBL65	ΔilvD	5x
BBS84	Mix – 11	BBL62	ΔhisG	1x
BBS85	Mix – 11	BBL62	ΔhisG	5x
BBS86	Mix – 11	BBL59	ΔcysH	1x
BBS87	Mix – 11	BBL59	ΔcysH	5x

Appendix Table S3. Summary of all selections. The first column gives the name of the sample collected (BBS###). The second column describes which fragment libraries were transformed. The third column gives a reference to the reference t_0 sample name (BBL###). The fourth column describes the knockout of the auxotrophic strain that was being transformed. The fitth and final column describes the aTc induction level.

E. coli BW25113 into ΔthrB			
barcode	Reads	Fraction	contained genes
CACTAGTTGAAGTGTGTGGG	4359217	0.998328187	**BW25113_RS00015 (thrB - homoserine kinase)** , BW25113_RS00020 (thrC - threonine synthase)
TTGCAAAATAAATCCTTATT	3396	0.000777737	BW25113_RS16045 (YgjU - serine symporter), BW25113_RS16050 (YgjV - inner membrane protein)
GCATGTTGCGGTGAAATAAG	3191	0.000730788	NA
GCGCCTACTTACGGCTATTG	419	9.60E-05	NA
TTATTTTCGTAAGTTGTGCG	122	2.79E-05	TK06_RS20840, TK06_RS20845
GTATGATCAGCGTCGCTGGG	84	1.92E-05	BW25113_RS17035 (smf), BW25113_RS17040 (fms), BW25113_RS17045 (ftm)
GTGCAGGGCAGAGACATTGT	45	1.03E-05	NA
TTCTGGTCTCGTGGGAGCAC	33	7.56E-06	BDW16_RS16065,BDW16_RS16070,BDW16_RS16075, BDW16_RS16080,BDW16_RS16085
GTTAGCTCGGGACAGACAAA	10	2.29E-06	TK06_RS20840, TK06_RS20845

Appendix Table S4. *First of three tests for liquid culture selection, Escherichia coli BW25113 into ΔthrB.* The table shows the barcode sequence, the number of reads that it accounted for, the fraction of the total number of reads the barcode accounts for in the experiment, and the contained genes, with the expected complementing gene bolded.

Sphingomonas koreensis into ΔthrB			
TTCTGGTCTCGTGGGAGCAC	4504153	0.977960804	BDW16_RS16065 (pirin), BDW16_RS16070 (LysR), BDW16_RS16075 (ribonuclease) ,**BDW16_RS16080 (homoserine kinase)**, BDW16_RS16085 (reductase)
GCAACTTGTAGTCAGGAAAG	101349	0.022005325	NA
TTATTTTCGTAAGTTGTGCG	103	2.24E-05	TK06_RS20840, TK06_RS20845
CACTAGTTGAAGTGTGTGGG	26	5.65E-06	BW25113_RS00015,BW25113_RS00020
GTTAGCTCGGGACAGACAAA	11	2.39E-06	TK06_RS20840,TK06_RS20845
AAATAGTTTTGAGGGGTCCG	4	8.68E-07	NA
GCGGGGCTAGTCTAAAGCAG	4	8.68E-07	NA
GGCAAGTGTGTTATGCGTGG	4	8.68E-07	NA
GTCAGTTGAGCCTCGCTGCG	4	8.68E-07	NA

Appendix Table S5. *Second of three tests of liquid culture outgrowth, Sphingomonas koreensis JSS26 into ΔthrB.* The table shows the barcode sequence, the number of reads that it accounted for, the fraction of the total number of reads the barcode accounts for in the experiment, and the contained genes, with the expected complementing gene bolded.

Pseudomonas fluorescens FW300-N2E2 into ΔthrB			
TTATTTTCGTAAGTTGTGCG	4004179	0.846429397	TK06_RS20840 (DUF2782) ,**TK06_RS20845 (homoserine kinase)**
GTTAGCTCGGGACAGACAAA	725435	0.153347168	TK06_RS20840 (DUF2782) ,**TK06_RS20845 (homoserine kinase)**
GTGTTCTTGTAACTCTGATT	969	0.000204834	TK06_RS30190
CACTAGTTGAAGTGTGTGGG	41	8.67E-06	BW25113_RS00015,BW25113_RS00020
TTCTGGTCTCGTGGGAGCAC	35	7.40E-06	BDW16_RS16065,BDW16_RS16070,BDW16_RS16075, BDW16_RS16080,BDW16_RS16085
TATGCAACGGTATTGCTACT	6	1.27E-06	NA
AACAGGTGCGAGGCGTCTTT	2	4.23E-07	TK06_RS23385
GATCACCGACTTTTATACAC	2	4.23E-07	NA
GATTTTAAATTTGCCGGCGA	2	4.23E-07	NA

Appendix Table S6. *Third of three tests of liquid culture outgrowth, Pseudomonas fluorescens FW300-N2E2 into ΔthrB.* The table shows the barcode sequence, the number of reads that it accounted for, the fraction of the total number of reads the barcode accounts for in the experiment, and the contained genes, with the expected complementing gene bolded.

organism	library	hit	number
Escherichia coli BW25113	pBWBHTC1	TRUE	18
Escherichia coli BW25113	pBWBHTC1	FALSE	1
Pedobacter sp. FW305-3-2-15-E-R2A2	pBWBHTC10	TRUE	2
Pedobacter sp. FW305-3-2-15-E-R2A2	pBWBHTC10	FALSE	16
Acidovorax sp. FHTAMBA	pBWBHTC11	TRUE	11
Acidovorax sp. FHTAMBA	pBWBHTC11	FALSE	7
Pseudomonas fluorescens FW300-N2E2	pBWBHTC2	TRUE	15
Pseudomonas fluorescens FW300-N2E2	pBWBHTC2	FALSE	7
Sphingomonas koreensis JSS26; DSMZ 15582	pBWBHTC3	TRUE	5
Sphingomonas koreensis JSS26; DSMZ 15582	pBWBHTC3	FALSE	12
Bacillus subtilis 168	pBWBHTC4	TRUE	10
Bacillus subtilis 168	pBWBHTC4	FALSE	9
Bacteroides thetaiotaomicron VPI-5482	pBWBHTC5	TRUE	8
Bacteroides thetaiotaomicron VPI-5482	pBWBHTC5	FALSE	6
Lysobacter sp. FW306-1B-D06B	pBWBHTC6	TRUE	5
Lysobacter sp. FW306-1B-D06B	pBWBHTC6	FALSE	13
Xylophilus sp. GW821-FHT01B05	pBWBHTC7	TRUE	2
Xylophilus sp. GW821-FHT01B05	pBWBHTC7	FALSE	18
Rhodanobacter sp. FW104-10B01	pBWBHTC8	TRUE	4
Rhodanobacter sp. FW104-10B01	pBWBHTC8	FALSE	15
Rhodoferax lacus GW822-FHT02A01	pBWBHTC9	TRUE	3
Rhodoferax lacus GW822-FHT02A01	pBWBHTC9	FALSE	16

Appendix Table S7. *Positive control return per genome.* The table shows the number of returned true positives and false negatives per genome for genes predicted to complete missing function.

Background	Source	locus_tag	protein_id	uniprotId	Expected	Comments
aroA	*Bacteroides thetaiotaomicron* VPI-5482	BT_RS11065	WP_162303115.1	Q8A5Q2	yes	35% identical to the *Vibrio cholerae* enzyme (Q9KRB0)
aroE	*Sphingomonas koreensis* JSS26; DSMZ 15582	BDW16_RS10815	WP_066578130.1	A0A2M8WD96	yes	Also known as Ga0059261_2194; RB-TnSeq data confirms that mutants are auxotrophic
hisC	*Acidovorax* sp. FHTAMBA	AAFF19_05770	XAH19868.1	A0A2R7PAQ8	yes	32% identical to HisC from *Caldanaerobacter* (Q8R5Q4); considered expected because it is in an operon with histidine synthesis genes
hisC	*Rhodoferax sp.* GW822-FHT02A01	AAGF34_01100	XAF49772.1	NA	no	31% identical to HisC from *Caldanaerobacter* (Q8R5Q4); is in an operon with histidine synthesis genes
metB	*Bacillus subtilis* 168	BSU_11870	NP_389069.1	A0A9Q4E7A0	no	This is the *B. subtilis* O-acetylhomoserine sulfhydrylase (MetI); it was previously reported to complement metB- *E. coli*, even though *in vitro*, the enzyme has no detectable activity on O-succinylhomoserine (PMID:11832514)
proB	*Pedobacter sp*. FW305-3-2-15-E-R2A2	AAFF35_11135	WZV14794.1	A0A1H0J514	yes	35% identical to CA265_RS20855, whose mutants are auxotrophic (RB-TnSeq data)
thrB	*Bacillus subtilis* 168	BSU_32240	NP_391104.1	A0AA96UM70	yes	Complementation was previously demonstrated (PMC1167255) but this information is not in the curated databases
trpA	*Pedobacter sp*. FW305-3-2-15-E-R2A2	AAFF35_06155	WZV13815.1	NA	yes	38% identical to the budding yeast enzyme (P00931)
trpA	*Acidovorax sp*. FHTAMBA	AAFF19_07375	XAH20166.1	H0BV16	yes	66% identical to RS_RS09955, whose mutant is auxotrophic for tryptophan (PMC8510521); this information is not in the curated databases
trpA	*Rhodanobacter denitrificans* FW104-10B01	LRK54_RS01680	WP_027489804.1	M4NLA4	yes	39.7% identical to the maize enzyme (B2Y0K4)

Appendix Table S8. *Diverged hits.* The table shows the knock out background for which the hit was identified, the source genome the fragment came from, the locus tag, protein_id and uniprot id of the identified open reading frame. We catalog whether or not the gene was expected based on our criteria in the manuscript and provide a description to what known gene it was homologous to and the percentage homology.

KO	locus_tag	protDesc	curated	protein_id	uniprotId	Genome	t0set	Validated
cysA	BSU_15580	sulfate permease	cysA*	NP_389441.1	A0A6M4JJU8	*Bacillus subtilis* (168)	Mix_11.cysA	Yes
cysA	TK06_RS10770	sulfite exporter TauE/SafE family protein	cysA*	WP_063322060.1	A0A1K1T875	*Pseudomonas fluorescens* (FW300-N2E2)	Mix_11.cysA	Yes
hisC	TK06_RS12685	histidinol-phosphate transaminase	NA	WP_063322357.1	A0A159ZZQ5	*Pseudomonas fluorescens* (FW300-N2E2)	Mix_11.hisC	Yes
metB	LRK54_RS05660	PLP-dependent aspartate aminotransferase family protein	NA	WP_027489953.1	A0A368KK22	FW104-10B01, *Rhodanobacter denitrificans*	pBWBHTC8.metB	Yes
ppc	BW25113_RS18290	C4-dicarboxylate transporter DctC	NA	WP_000858214.1	D3H0Y2	*Escherichia coli* BW25113	Mix_11.ppc	Crossfed
ppc	BDW16_RS00990	dicarboxylate/amino acid:cation symporter	NA	WP_066575250.1	A0A1L6JC14	*Sphingomonas koreensis*	Mix_11.ppc	Crossfed
ppc	TK06_RS28425	dicarboxylate/amino acid:cation symporter	NA	WP_063324741.1	A0A160A458	*Pseudomonas fluorescens* (FW300-N2E2)	Mix_11.ppc	Crossfed
trpA	BT_RS23500	TrpB-like pyridoxal phosphate-dependent enzyme	trpB2	WP_008760379.1	D7IA57	*Bacteroides thetaiotaomicron*	Mix_11.trpA	Crossfed
pheA	LRK54_RS17455	MFS transporter	NA	WP_063090425.1	M4NEK0	FW104-10B01, *Rhodanobacter denitrificans*	Mix_11.pheA	No
pheA	TK06_RS20405	MFS transporter	NA	WP_063323551.1	A0A160A0V0	*Pseudomonas fluorescens* (FW300-N2E2)	Mix_11.pheA	No
proB	BW25113_RS20525	phosphoenolpyruvate carboxylase	ppc	WP_001005586.1	C5A0C2	*Escherichia coli* BW25113	Mix_11.ppc	No
proB	TK06_RS22845	dihydroxy-acid dehydratase	ilvD	WP_063323942.1	A0A160A1W0	*Pseudomonas fluorescens* (FW300-N2E2)	Mix_11.proB	No
pyrD	TK06_RS26365	SDR family oxidoreductase	NA	WP_063324432.1	A0A165ZNW9	*Pseudomonas fluorescens* (FW300-N2E2)	Mix_11.pyrD	No
cysH	BSU_32380	putative transporter	NA	NP_391118.1	A0A6H0H720	*Bacillus subtilis* (168)	Mix_11.cysH	No

Appendix Table S9. *Follow Up tests.* List of all unexpected hits that were followed up upon as individually cloned and tested, the contexts in which they were found, source genomes, protein identifiers, and the outcome of the follow up experiments (validated, cross feeding, or not validated).

Appendix References

Price MN & Arkin AP (2017) PaperBLAST: Text Mining Papers for Information about Homologs. *mSystems* 2

Price MN, Deutschbauer AM & Arkin AP (2020) GapMind: Automated Annotation of Amino Acid Biosynthesis. *mSystems* 5

Price MN, Wetmore KM, Waters RJ, Callaghan M, Ray J, Liu H, Kuehl J V., Melnyk RA, Lamson JS, Suh Y, *et al* (2018) Mutant phenotypes for thousands of bacterial genes of unknown function. *Nature* 557: 503–509
