# Supplementary material for: High-throughput protein characterization by complementation using DNA barcoded fragment libraries
Source: Mol Syst Biol. 2024 Oct 7;20(11):5. doi: 10.1038/s44320-024-00068-z (PMC11535334; doi:10.1038/s44320-024-00068-z)
Supplement: Supplementary file 11 — Expanded View Figures [file 44320_2024_68_MOESM11_ESM.pdf]

Expanded View Figures

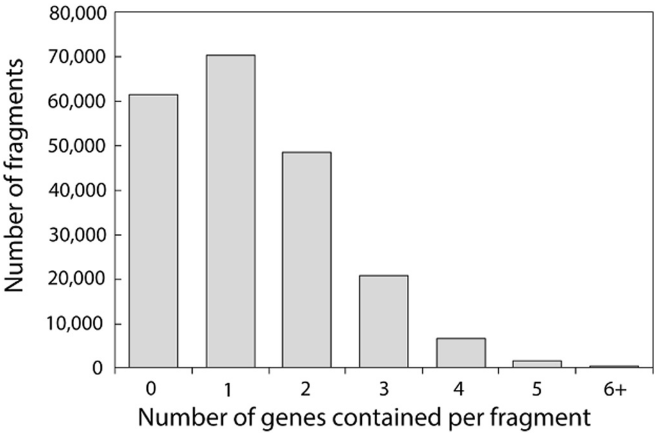

Figure EV1. Number of genes contained per fragment across all 11 libraries.

The *x* axis shows the number of genes per fragment. The *y* axis shows the number of fragments per category.

 © The Author(s)

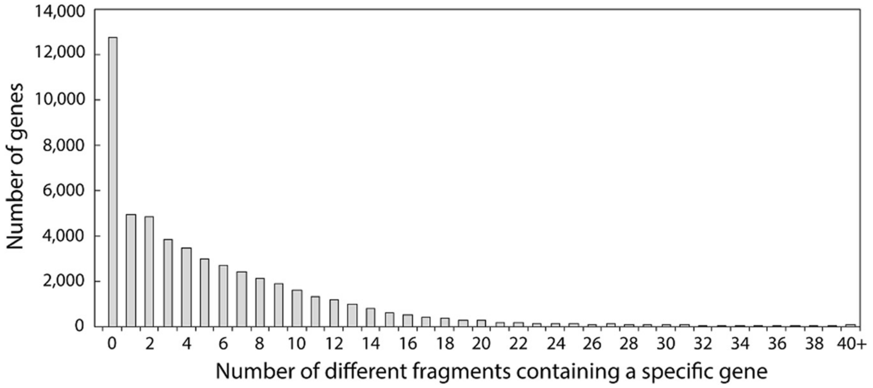

Figure EV2. Number of fragments covering each gene across all 11 libraries.

The *x* axis shows the number of different unique fragments that cover a given gene. The *y* axis shows the number of genes covered per category (i.e., covered by 1 fragment, by 2 fragments, etc.).
